# Deletion of fatty acid amide hydrolase reduces lyso-sulfatide levels but exacerbates metachromatic leukodystrophy in mice

**DOI:** 10.1016/j.jbc.2021.101064

**Published:** 2021-08-08

**Authors:** Claudia Yaghootfam, Bernd Gehrig, Marc Sylvester, Volkmar Gieselmann, Ulrich Matzner

**Affiliations:** 1 Medical Faculty, Institute of Biochemistry and Molecular Biology, University of Bonn, Bonn, Germany; 2 Medical Faculty, Core Facility Mass Spectrometry, Institute of Biochemistry and Molecular Biology, University of Bonn, Bonn, Germany

**Keywords:** lysosomal storage disease, metachromatic leukodystrophy, glycosphingolipid, lyso-sulfatide, endo-N-deacylase, fatty acid amide hydrolase, ACER1, alkaline ceramidase-1, ACY1, aminoacylase, AEA, anandamide, AMCAA, 7-amino-4-methyl coumarin arachidonoyl amide, ASA, arylsulfatase A, ASAH1, acid ceramidase, ASAH2, neutral ceramidase, BSA, bovine serum albumin, CPVL, carboxypeptidase A, CST, cerebroside sulfotransferase, CTSB, cathepsin B, CTSH, cathepsin H, CTSS, cathepsin S, CTSZ, cathepsin Z, DPEP1, dipeptidase I, FAAH, fatty acid amide hydrolase, GALC, galactosylceramidase, GSL, glycosphingolipid, hASA, human ASA, HPLC, high-performance liquid chromatography, mASA, murine ASA, MIP-1α, macrophage inflammatory protein-1α, MLD, metachromatic leukodystrophy, NAAA, N-acylethanolamine-hydrolyzing acid amidase, NBD, nitrobenzoxadiazole, SCDase, sphingolipid ceramide N-deacylase, SCPEP1, carboxypeptidase C, sLDL, synthetic low-density lipoprotein, tg, transgenic, TLC, thin-layer chromatography

## Abstract

An inherited deficiency of arylsulfatase A (ASA) causes the lysosomal storage disease metachromatic leukodystrophy (MLD) characterized by massive intralysosomal storage of the acidic glycosphingolipid sulfatide and progressive demyelination. Lyso-sulfatide, which differs from sulfatide by the lack of the N-linked fatty acid, also accumulates in MLD and is considered a key driver of pathology although its concentrations are far below sulfatide levels. However, the metabolic origin of lyso-sulfatide is unknown. We show here that ASA-deficient murine macrophages and microglial cells express an endo-N-deacylase that cleaves the N-linked fatty acid from sulfatide. An ASA-deficient astrocytoma cell line devoid of this activity was used to identify the enzyme by overexpressing 13 deacylases with potentially matching substrate specificities. Hydrolysis of sulfatide was detected only in cells overexpressing the enzyme fatty acid amide hydrolase (FAAH). A cell-free assay with recombinant FAAH confirmed the novel role of this enzyme in sulfatide hydrolysis. Consistent with the *in vitro* data, deletion of FAAH lowered lyso-sulfatide levels in a mouse model of MLD. Regardless of the established cytotoxicity of lyso-sulfatide and the anti-inflammatory effects of FAAH inhibition seen in mouse models of several neurological diseases, genetic inactivation of FAAH did not mitigate, but rather exacerbated the disease phenotype of MLD mice. This unexpected finding was reflected by worsening of rotarod performance, increase of anxiety-related exploratory activity, aggravation of peripheral neuropathy, and reduced life expectancy. Thus, we conclude that FAAH has a protective function in MLD and may represent a novel therapeutic target for treatment of this fatal condition.

Glycosphingolipids (GSLs) consist of the amino alcohol sphingosine or a related sphingoid base that is linked to a fatty acid *via* an amide bond and to a saccharide head group *via* an O-glycosidic linkage (1). The saccharide moiety of the acidic GSL sulfatide (3-O-sulfogalactosylceramide), a key component of myelin sheaths, is a sulfated galactosyl residue. Sulfatide is degraded in the lysosomal compartment by an ordered sequence of reactions, starting with desulfation through arylsulfatase A (ASA; EC 3.1.6.8), then removal of the galactosyl residue through galactosylceramidase (GALC; EC 3.2.1.46), and eventually cleavage of the N-linked fatty acid through acid ceramidase (ASAH1; EC 3.5.1.23). The hydrolytic products, sulfate, galactose, fatty acid, and sphingosine, are exported from the lysosome for reutilization. Lack of either ASA, GALC, or ASAH1 results in a fatal lysosomal storage disease designated as metachromatic leukodystrophy (MLD; OMIM 250100), globoid cell leukodystrophy (Krabbe disease; OMIM 245200), and Farber disease (OMIM 228000), respectively (2). They are characterized by intralysosomal accumulation of sulfatide, galactosylceramide (except CNS), and ceramide, respectively.

Lyso-sulfatide emerges as a minor storage product in the nervous system of MLD patients and mouse models of the disease (3). It differs from sulfatide by the absence of the N-linked fatty acid. The well-established cytotoxicity of this and related lyso-GSLs gave rise to a hypothesis that the lyso-forms, rather than the parental GSLs themselves, are key drivers of pathology in GSL storage diseases (4). Micromolar concentrations of lyso-sulfatide, for example, inhibit protein kinase C, cytochrome c oxidase activity, and migration of neuronal precursor cells *via* calcium-mediated process collapse (5, 6, 7). The origin of lyso-GSLs has been debated for a long time. Recent genetic and pharmacological data suggest that ASAH1 has a broader substrate specificity than initially supposed and deacylates not only ceramide, but also certain GSLs (8). For sulfatide, however, cleavage by ASAH1 could not be demonstrated so far. Alternatively, an anabolic source of lyso-sulfatide due to the accidental sulfation of psychosine (instead of galactosylceramide) by the galactose-3-sulfotransferase-1 has been proposed (9). On the other hand, two independent studies indicated degradation of sulfatide by unidentified nonlysosomal hydrolase(s) that in retrospect, although not proven, might be enzyme(s) with endo-N-deacylase activity (10, 11).

The main challenge in identifying such an endo-N-deacylase is that it might accept and convert sulfatide only as a secondary substrate and with a low catalytic efficiency k_cat_/K_m_. Analytical tools of high sensitivity will be required to demonstrate such a side reaction that might become physiologically relevant only if sulfatide accumulates to supraphysiological levels. Furthermore, the sought enzyme could be nonlysosomal, as lysosomally stored GSLs might shuttle to other cellular membranes *via* transporters (1) or interorganelle membrane contact sites (12). Here we report *in vitro* and *in vivo* experiments leading to the identification of a so far unrecognized sulfatide-hydrolyzing activity of fatty acid amide hydrolase (FAAH), an endoplasmic reticulum-localized amidase renowned for its function in the endocannabinoid metabolism.

## Results

### Optimization of sulfatide loading by translipofection

**Figure 1:**
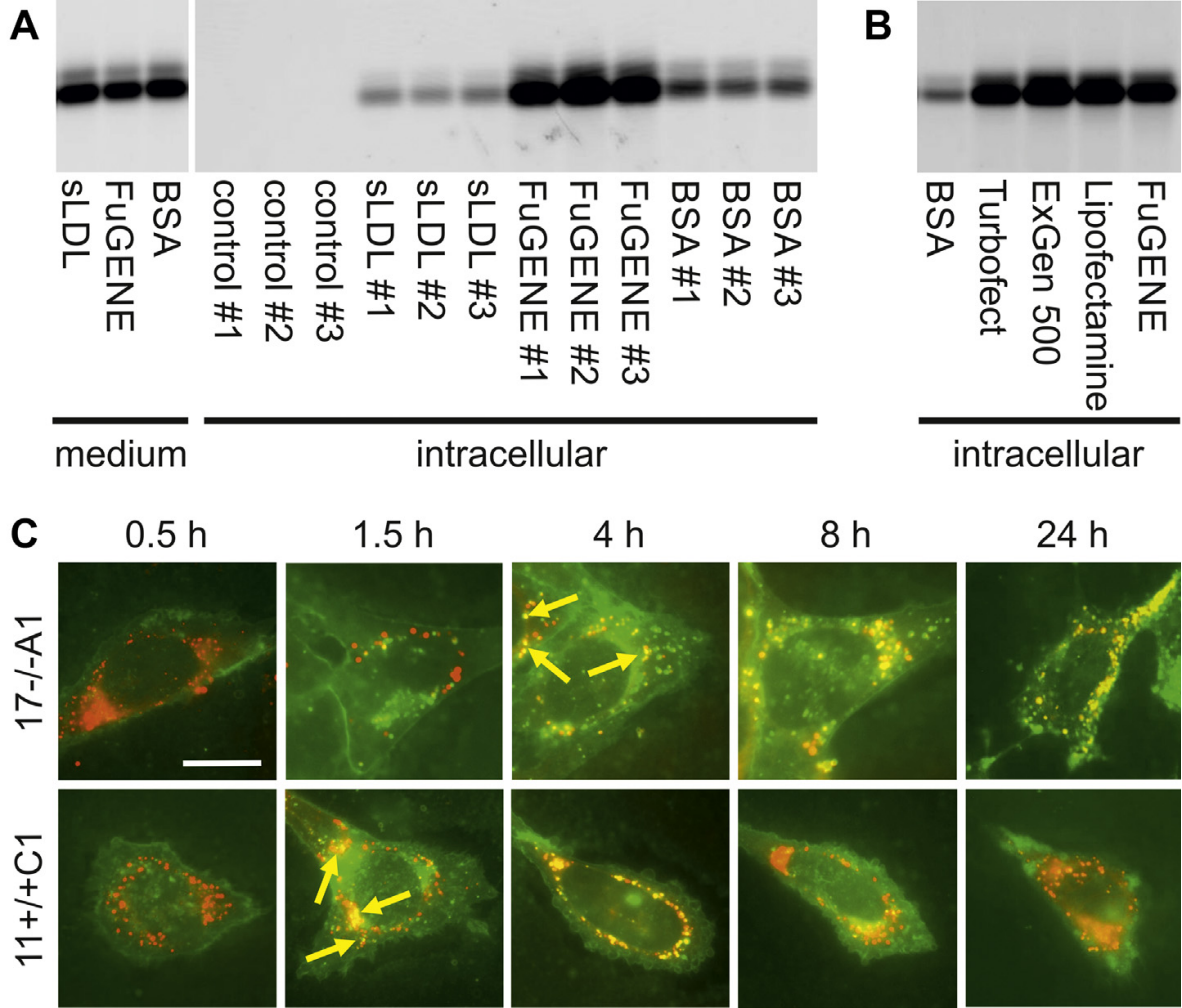
**Loading of astrocytoma cells with fluorescently labeled sulfatide.***A*, cellular uptake of NBD sulfatide by ASA-deficient 17−/−A1 cells using three different loading protocols. For each condition incubation medium (*left panel*) was evenly distributed to three dishes (#1, 2, 3) that were analyzed separately (*right panel*). Fluorescence scanning of cellular lipid extracts separated by TLC reveals that *translipofection* with FuGENE HD transfection reagent is more efficient than sLDL- and BSA-mediated internalization. *B*, uptake of NBD sulfatide after preincubation with BSA, cationic polymers (Turbofect, ExGen 500), and cationic lipids (Lipofectamine 2000, FuGENE HD), respectively. *C*, fluorescence microscopy of ASA-deficient 17−/−A1 astrocytoma cells (*upper panel*) and wild-type 11+/+C1 cells (*lower panel*) at different times after *translipofection* of BODIPY 500/510-labeled sulfatide (*green*). Before sulfatide loading, cells were preincubated with the Golgi marker BODIPY-TR-ceramide (*red*). Appearing colocalizations (*yellow*) of BODIPY 500/510 and BODIPY-TR are indicated by *yellow arrows*. All cells are shown at identical magnification. The scale bar represents 10 μm.

Cultured cells take up fluorescently or radioactively labeled GSLs from the medium and target them to lysosomes for degradation. Such cell loading assays are an established means to diagnose GSL storage diseases and to analyze the intracellular degradation pathways of GSLs. To increase the sensitivity of sulfatide loading assays, we exploited the negative charge of its sulfate group and tested cationic cell transfection reagents on their ability to promote cellular uptake. Complexation of NBD-labeled sulfatide with FuGENE HD proved to be more efficient with regard to internalization efficacy and cell viability than other cationic transfection reagents and previously published methods of complexation with BSA or packaging in synthetic lipoprotein particles (Fig. 1, *A* and *B*). Compared with the classical BSA complexation method, uptake of NBD-sulfatide was three- to four-fold increased. We designate this novel loading method as *translipofection*.

To evaluate the dissemination of fluorescently labeled sulfatide and its breakdown products on a subcellular level, wild-type and ASA-deficient cells were *translipofected* and analyzed by life-cell fluorescence microscopy. For this purpose we used two astrocytoma cell lines derived from wild-type mouse brain (11+/+C1) and ASA-knockout mouse brain (17−/−A1), respectively (13). To circumvent the low photostability of NBD, making long-term *in vivo* imaging difficult, sulfatide carrying an N-linked fatty acid with a more stable BODIPY 500/510 fluorophore (green) was synthesized (14). Cells were costained with the Golgi marker BODIPY-TR-ceramide (red). In wild-type 11+/+A1 cells, the green fluorescence of *translipofected* BODIPY 500/510-labeled sulfatide shifted from perinuclear vesicular structures, probably representing endosomes and lysosomes, through the Golgi apparatus to diffusely stained cellular compartments (Fig. 1*C*, lower panel). This redistribution can be explained by intralysosomal degradation of sulfatide to ceramide, its cleavage by ASAH1, export of the liberated BODIPY 500/510-labeled fatty acid to the ER, and reincorporation into complex phospho- and sphingolipids passing the Golgi apparatus to various cellular membranes. Due to the lack of ASA, BODIPY-labeled sulfatide cannot be hydrolyzed in 17−/−A1 cells causing accumulation of the green fluorescence in the endosomal/lysosomal compartment (Fig. 1*C*, upper panel; timepoint 0.5 and 1.5 h). Notably, also in this condition BODIPY 500/510-labeled sulfatide (or sulfatide-derived lipids) eventually escaped from lysosomal entrapment and reached the Golgi apparatus. Compared with wild-type cells, this redistribution was, however, substantially retarded and Golgi localization of BODIPY 500/510 could be seen 4 h after *translipofection* for the first time. Similar kinetics of redistribution were obtained for NBD-labeled sulfatide (not shown).

### Translipofection of human fibroblasts

**Figure 2:**
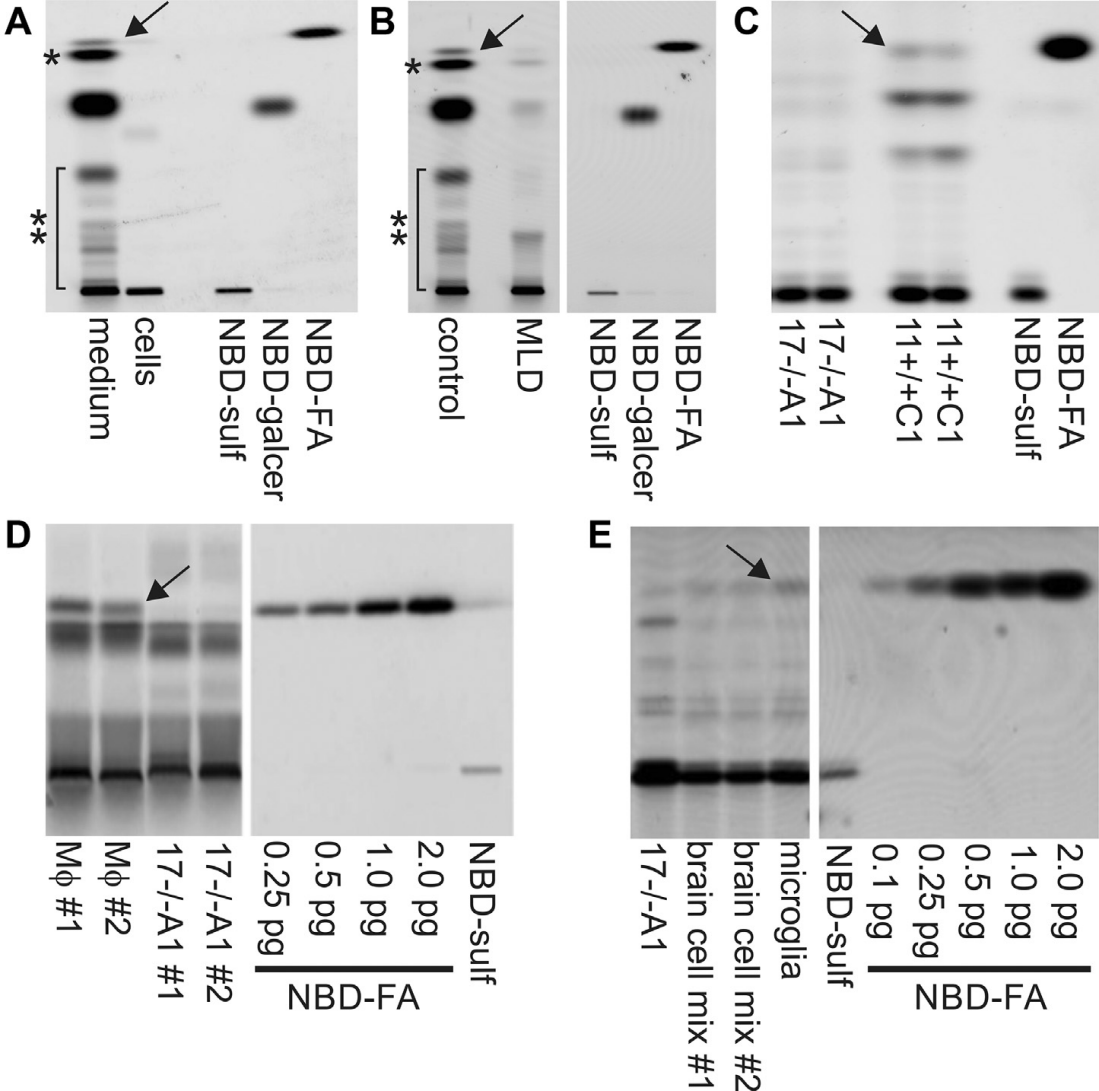
***Translipofection* of cells with NBD-sulfatide carrying an N-linked fatty acid with terminal (omega) fluorophore.** Cells were loaded for 6 h and subsequently cultured in the absence of NBD-sulfatide for 18 h. Fluorescing breakdown products of sulfatide and newly synthesized lipids with reincoporated NBD-fatty acid were visualized by fluorescence scanning following TLC separation of lipid extracts. NBD-labeled sulfatide (NBD-sulf), galactosyl ceramide (NBD-galcer) and fatty acid (NBD-FA) were coseparated as standards. Lanes from two different sections of the same TLC plate have been combined in (*B*), (*D*), and (*E*), respectively. The *vertical white lines* mark the splice borders. *A*, lipid extracts from cells and medium of *translipofected* wild-type human fibroblasts. Note that NBD-labeled fatty acid (*arrow*), ceramide (∗), and newly synthesized phosphoglycerolipids and sphingolipids (∗∗) are efficiently released to the medium within the chase period of 18 h. *B*, fibroblasts from a juvenile MLD patient with <5% residual ASA activity compared to control fibroblasts as indicated. Note that the MLD fibroblasts convert some NBD-sulfatide. *C*, murine astrocytoma cells derived from an ASA knockout mouse (17−/−A1) and a wild-type control mouse (11+/+C1), respectively. The ASA-expressing, but not the ASA-deficient cells hydrolyze NBD sulfatide. *D*, in contrast to 17−/−A1 cells (dishes #1 and #2), two preparations of ASA-deficient primary murine macrophages prepared from an ASA knockout mouse (MΦ #1 and MΦ #2) efficiently liberate NBD-fatty acid (*arrow*) from NBD sulfatide. In this experiment macrophages and 17−/−A1 cells were cultured in Fischer's medium with phenol red producing increased background fluorescence. *E*, inefficient release of NBD-fatty acid by two mixed primary cultures of mouse brain cells (brain cell mix #1 and #2) is enhanced after enrichment of microglial cells (*arrow*). Extracts of 17−/−A1 cells were loaded as a negative control.

To characterize the fluorescing intermediates biochemically, lipid extracts of *translipofected* cells were separated by thin layer chromatography (TLC) and the chromatograms were scanned with a FLA-5100 fluorescence scanner showing a limit of detection around 10 fmol NBD per band. In a first experiment, wild-type human fibroblasts were *translipofected*. After 6 h, sulfatide (undegraded), galactosylceramide, ceramide, and free fatty acids could be identified by coseparation of NBD-labelled standards (Fig. 2*A*). Liberated NBD-labeled fatty acids were also reincorporated into composite lipids representing sphingolipids and phosphoglycerolipids as suggested by their Rf values and differential resistance to mild alkaline hydrolysis (not shown). Interestingly, signals were much more intense in lipid extracts from the medium than in cellular extracts indicating efficient export of NBD-containing metabolites probably *via* ATP-binding cassette transporters (15). This allowed us to investigate the intracellular metabolism of NBD sulfatide by analyzing conditioned medium in all following experiments.

In a next experiment cells from a juvenile MLD patient with the disease-causing mutation p.P426L (“allele A”) (16) were *translipofected*. This mutation results in a residual ASA activity below 5% of normal. Importantly, signals for NBD-labeled galactosylceramide, ceramide, and fatty acid, albeit being very faint, were still discernible (Fig. 2*B*, second lane). Thus, *translipofection* of NBD sulfatide allows to measure also a low residual turnover of sulfatide.

### Sulfatide-hydrolyzing endo-N-deacylase activity of murine microglia and macrophages

Next, we compared the sulfatide catabolism of the two murine astrocytoma cell lines 11+/+C1 and 17−/−A1. ASA-expressing 11+/+C1 cells were able to liberate the NBD-labeled fatty acid (Fig. 2*C*, arrow) indicating sulfatide degradation. In contrast, no fatty acid signal was discernible for 17−/−A1 cells. This reflects a complete blockade of the sulfatide catabolism in these ASA-deficient astrocytoma cells.

**Figure 3:**
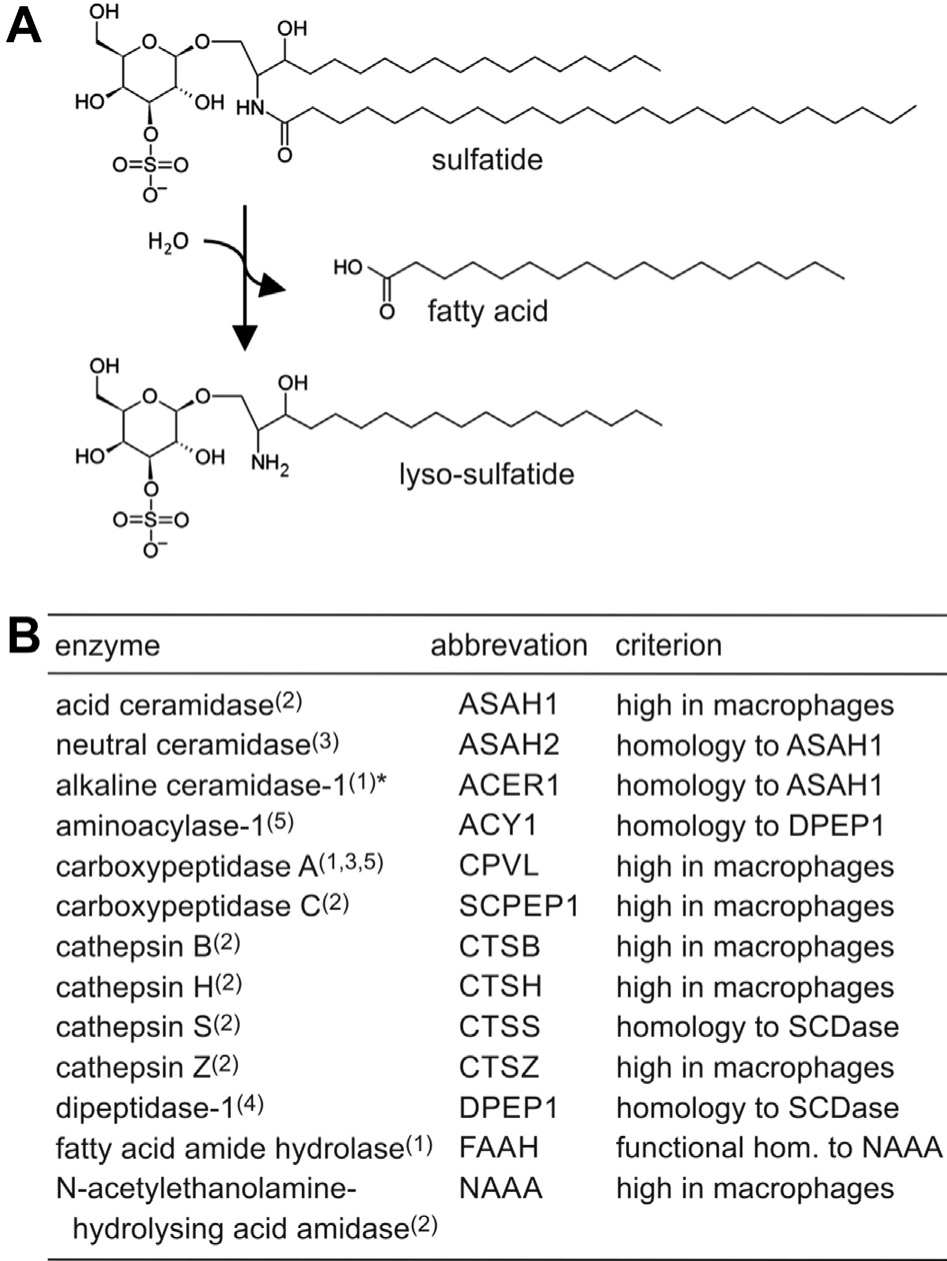
**Endo-N-deacylation of sulfatide.***A*, reaction scheme illustrating direct deacylation of sulfatide to lyso-sulfatide and free fatty acid as suggested by loading assays of macrophages and microglial cells. *B*, enzyme candidates with potential endo-N-deacylase activity toward sulfatide. The enzymes were selected from the literature as described in the text and summarized in the *right column*. *Superscript numbers* in the *left column* indicate the cellular localization: 1 – ER, 2 – lysosome, 3 – plasma membrane, 4 – extracellular, 5 – cytosol. *Asterisk*: also alkaline ceramidase-2 and -3 were selected as candidates, but full-length cDNAs were not available from public sources.

To investigate the sulfatide catabolism of ASA-deficient macrophages and microglial cells, we *translipofected* primary bone-marrow-derived macrophages and brain microglial cells isolated from ASA knockout mice. Surprisingly and in contrast to 17−/−A1 cells, both cell types released significant amounts of the NBD-labeled fatty acid (Fig. 2, *D* and *E*, arrows). Furthermore, NBD-galactosylceramide representing a prominent catabolic intermediate of the lysosomal degradative pathway in wild-type cells (Fig. 2, *A*–*C*) was undetectable. Taken together, these observations pointed toward an ASA-independent pathway of sulfatide degradation in macrophages and microglial cells involving direct deacylation (Fig. 3*A*).

### Selection of potential sulfatide-hydrolyzing endo-N-deacylases

In an attempt to identify the sulfatide-hydrolyzing endo-N-deacylase activity of macrophages and microglial cells, published enzyme data were evaluated. Hydrolases with possibly matching substrate specificities acting on carbon-nitrogen bonds of peptides (E.C. 3.4) or other linear amides (E.C. 3.5.1) were selected from the BRENDA database (https://www.brenda-enzymes.org/) by applying one of the following three criteria. (i) lysosomal localization (according to Ref (17)) and high expression in cells of the monocyte/macrophage/microglial lineage (http://genevisible.com). Enzymes with complex substrate specificity requirements that could not be brought into compliance with the molecular structure of sulfatide were arbitrarily omitted. This led to the elimination of, *e.g.*, cathepsins with endopeptidase activity (see Tables S2–S4). (ii) Nonlysosomal enzymes being evolutionary related to the selected lysosomal enzymes or have a very similar substrate specificity. Proteases were omitted to reduce the number of candidates (see Table S5). (iii) Enzymes with sequence similarities to the putative active site of sphingolipid ceramide N-deacylase from Shewanella algae (amino acids 516–643) (18) or *Pseudomonas* sp. TK-4 (amino acids 119–186; https://patents.google.com/patent/US6821761B2), two bacterial endo-N-deacylases accepting complex GSLs as substrates. BlastP (https://blast.ncbi.nlm.nih.gov/Blast.cgi) was used to compare the active sites with human nonredundant protein sequences and revealed weak similarities with dipeptidase I (DPEP1, 24% identity) and cathepsin S (CTSS, 34% identity), respectively (Fig. S1). Figure 3*B* lists the 13 candidates resulting from the selection procedure. The full-length cDNAs of the candidates were cloned into the eukaryotic expression plasmid pcDNA3.

### Evaluation of enzyme candidates by translipofection of 17−/−A1 cells

**Figure 4:**
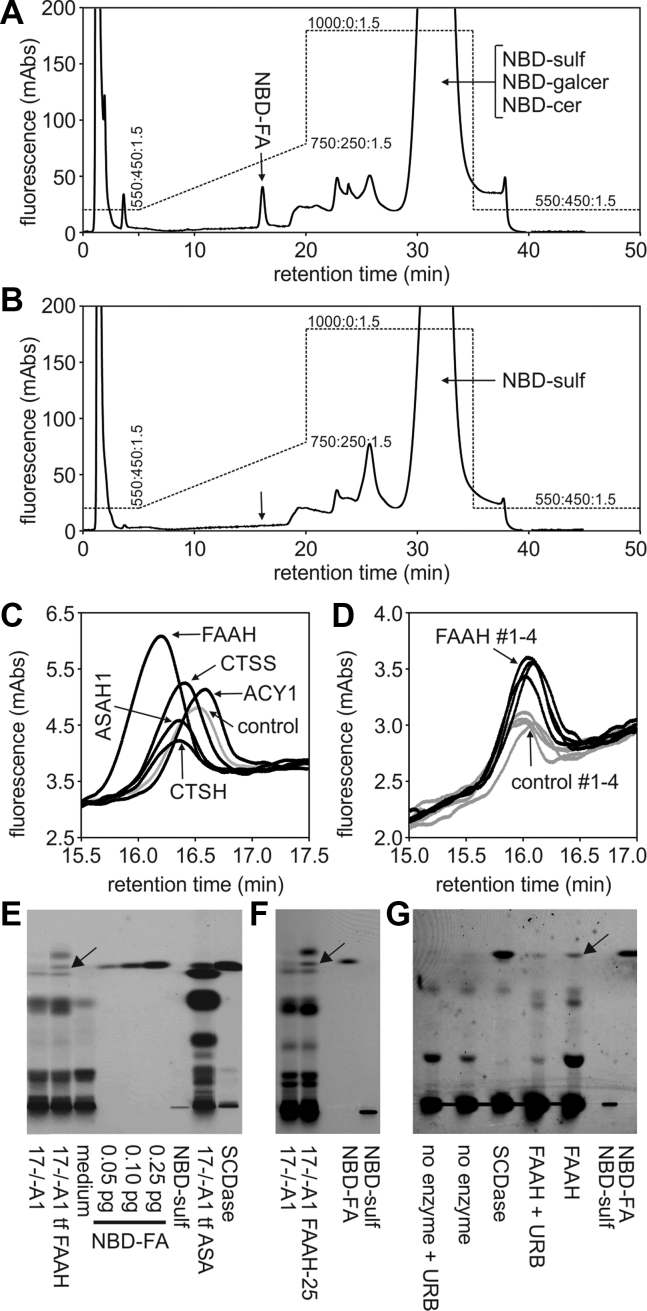
**Hydrolysis of *translipofected* NBD-sulfatide by FAAH.***A* and *B*, HPLC profiles of NBD-labelled lipids extracted from medium of *translipofected* 17−/−A1 cells. Cells that had been metabolically corrected by transfection of wild-type human ASA prior to loading (*A*) were compared with untransfected cells (*B*). NBD-labeled fatty acid (NBD-FA) is detectable only for the corrected cells (*arrow*). *C*, HPLC signals for NBD-fatty acids following transient transfection of 17−/−A cells with candidate enzymes as indicated. A representative experiment in which FAAH was compared with four other enzymes is shown. The UV profiles obtained in successive HPLC runs are superimposed. Only FAAH increased the background signal. *D*, HPLC signals for NBD-fatty acids in 17−/−A1 cells transfected with FAAH and empty expression vector as a control, respectively. Profiles of four technical replicates are shown. *E*, TLC of lipid extracts from medium of 17−/−A1 cells. A signal for NBD-fatty acid is visible after transient transfection of FAAH (tf FAAH, *arrow*), but not for untransfected cells. As a positive control 17−/−A1 cells were metabolically corrected by transfection of human ASA. As a further control NBD sulfatide was incubated with recombinant bacterial sphingolipid ceramide N-deacylase (SCDase) in the absence of cells. *F*, same experiment as shown in (*E*) with stably transfected 17−/−A1 cells (clone 17−/−A1 FAAH-25). *G*, cleavage of NBD sulfatide by recombinant human FAAH in a cell-free *in vitro* assay. The liberated fatty acid is indicated by an *arrow*. URB597 (“URB”) partially inhibits the reaction. Bacterial SCDase was used as a positive control. No fatty acid is seen in the absence of enzyme.

Because ASA-deficient 17−/−A1 astrocytoma cells are not capable to degrade NBD sulfatide (Fig. 2*C*), they could be utilized to evaluate alternate sulfatide-hydrolyzing pathways using transgenic expression of ectopic deacylases. For this purpose, 17−/−A1 cells were transiently transfected with the candidate enzymes (Fig. 3*B*), *translipofected* with NBD sulfatide, and the release of NBD fatty acid to the medium was analyzed by high-performance liquid chromatography (HPLC). As a control, 17−/−A1 cells were metabolically corrected by transfection of wild-type human ASA. Such ASA-expressing control cells released the NBD-fatty acid eluting from the HPLC column as a single peak with a retention time of ∼16 min (Fig. 4*A*). Confirming the TLC data, no such peak could be seen for uncorrected 17−/−A1 cells (Fig. 4*B*). When the sensitivity of the fluorescence detector was increased, a faint signal was, however, detectable also for mock-transfected 17−/−A1 cells (Fig. 4, *C* and *D*, control). When the 13 candidate enzymes were individually expressed in 17−/−A1 cells, none of them but one increased the signal for the NBD fatty acid compared with control cells transfected with empty vector. The exception was FAAH that increased the peak area by a factor of 2.01 ± 0.42 (mean ± SD) in five independent transfection experiments. Overexpression of ASAH1, on the contrary, had no effect (factor 1.12 ± 0.38). Liberation of the NBD fatty acid by FAAH-transfected cells was confirmed by TLC (Fig. 4*E*). We then generated 17−/− A1 cells stably transfected with either FAAH, or as a control, with ASAH1. Expression levels of single clones were analyzed with a fluorescence-based assay measuring deacylation of AMCAA (for FAAH) and western blotting (for ASAH1), respectively. The clone with the highest FAAH activity, called FAAH-25, released 2.40-fold more NBD fatty acid than control cells on average (Fig. 4*D*), whereas the clone with the highest ASAH1 expression showed no increase (factor 0.98, not shown). Increased release of NBD fatty acid by FAAH-25 cells could be independently demonstrated by TLC (Fig. 4*F*).

### *In vitro* activity of recombinant FAAH

To provide further evidence for a possible role of FAAH in the hydrolysis of sulfatide, NBD-labeled sulfatide was reacted with recombinant human FAAH in a cell-free *in vitro* assay. As a positive control, we used a bacterial SCDase that is known to hydrolyze the N-acyl linkage between fatty acids and sphingosine bases in various GSLs. Both, recombinant SCDase and, to a lower extent, also recombinant FAAH released significant amounts of NBD-labeled fatty acid from NBD sulfatide (Fig. 4*G*). Importantly, the competitive FAAH-inhibitor URB597 reduced liberation of the fatty acid in the FAAH-catalyzed reaction. The residual turnover of sulfatide in the presence of competitive amounts of inhibitor might be explained by the limited thermostability of URB597 possibly leading to a progressive decline of its inhibitory activity over the applied incubation time of 24 h.

### Lyso-sulfatide levels of FAAH-deficient MLD mice

**Figure 5:**
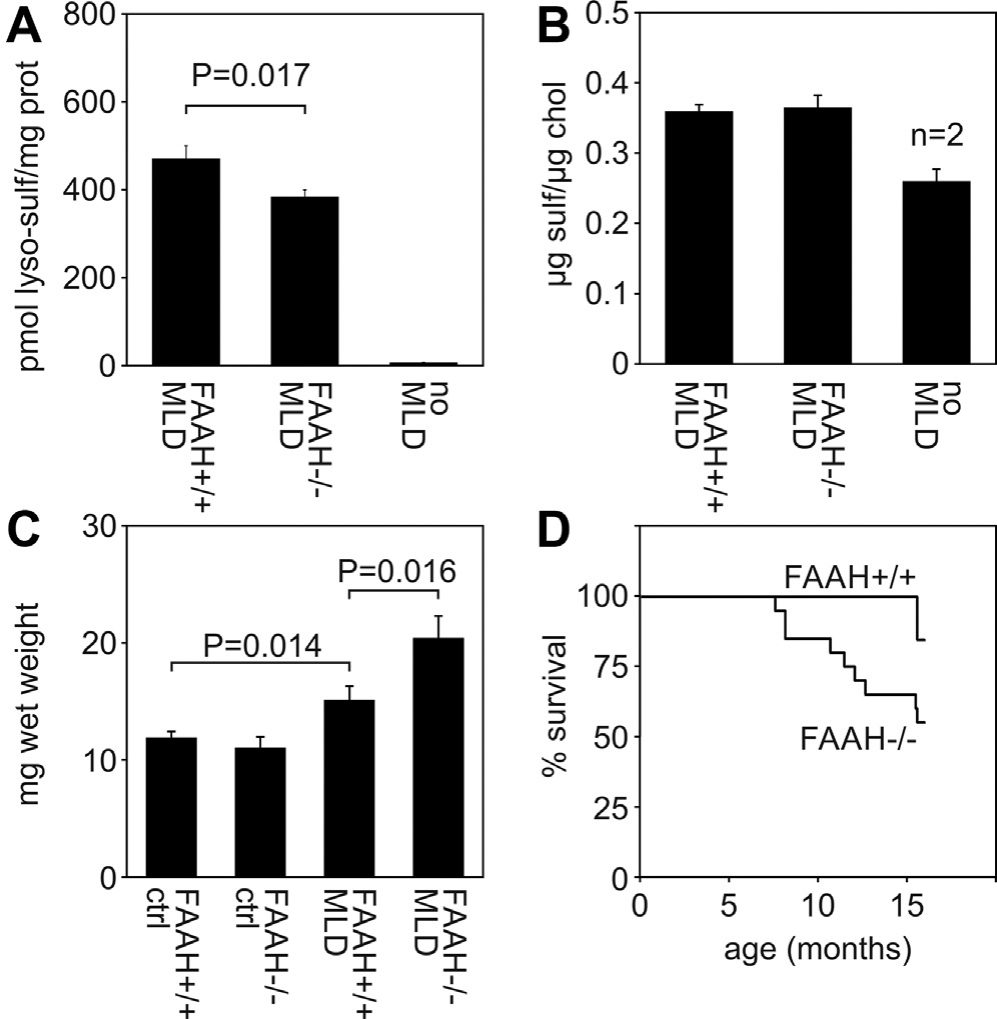
**Effects of FAAH deficiency on lipid profiles, peripheral neuropathy, and life expectancy.***A* and *B*, concentrations of lyso-sulfatide (*A*) and sulfatide (*B*) in total brain of 6 months old mice. Levels of the two lipids were normal in FAAH-deficient mice expressing ASA (not shown) that were combined with wild-type mice to group “no MLD”. Bars represent means ± SEM of the indicated experimental groups. Unless otherwise indicated, group sizes were 5 to 6 for MLD mice and three for control mice. Lyso-sulfatide concentrations of FAAH-expressing and FAAH-deficient MLD mice were compared using student’s *t* test and the corresponding *p*-value is indicated. *C*, wet weights of the sciatic nerves as a measure of hypertrophic neuropathy. Bars represent means ± SEM of n = 8 to 9 mice for MLD mice and 4 to 8 for control mice, respectively. The indicated groups of mice were compared by Student's *t* test and the *p*-values are indicated. *D*, life expectancy of two cohorts of FAAH-expressing and FAAH-deficient MLD mice, comprising 13 and 20 animals, respectively. Survivors were sacrificed at age 16 months. A comparison of the two Kaplan–Meier curves by a Gehan–Breslow–Wilcoxon test revealed significantly different life spans (*p* = 0.045).

The described experiments suggested that FAAH deacylates NBD sulfatide *in vitro*. To investigate if FAAH is also involved in the catabolism of (unlabeled) sulfatide *in vivo*, we constructed mice with a combined deficiency of ASA and FAAH. Furthermore, the mice were transgenic for the cerebroside sulfotransferase catalyzing the last step in the biosynthesis of sulfatide (19). Due to an increased synthesis and lysosomal storage of sulfatide, these mice develop more severe MLD-like disease than conventional ASA knockout mice. If sulfatide were deacylated to lyso-sulfatide by FAAH *in vivo*, FAAH-deficient MLD mice should produce less lyso-sulfatide than FAAH-expressing MLD mice. The quantification of lyso-sulfatide levels in brains of 6-month-old double-deficient mice revealed a significant decline by 18.4% (Fig. 5*A*), confirming this prediction and thus an *in vivo* function of FAAH in the sulfatide catabolism. FAAH deficiency did not affect sulfatide levels (Fig. 5*B*) being approximately two orders of magnitude higher than lyso-sulfatide levels (not shown).

### Peripheral neuropathy and life expectancy of FAAH-deficient MLD mice

MLD mice develop a hypertrophic neuropathy characterized by an increased deposition of collagen fibrils in the endoneurial space leading to a thickening of peripheral nerves (19). To evaluate a possible effect of FAAH on the caliber of the sciatic nerve, the nerves were dissected from 16-month-old mice and weighed. In ASA-expressing control mice, a deficiency of FAAH had no effect on the wet weights ranging between 11 and 12 mg on average (weight of the two nerves per mouse combined; Fig. 5*C*). Compared with the control mice, mean weights were, however, significantly increased to 15.1 mg in MLD mice (expressing FAAH) confirming the previous data for the phrenic nerve (19). If FAAH was eliminated in addition, the hypertrophic neuropathy was further aggravated and the wet weight of the two nerves per mouse reached 20.4 mg on average. Consequently, the existing hypertrophic neuropathy of MLD mice is intensified if FAAH is lacking.

MLD mice develop a paralysis of hindlimbs around age 16 months and their life expectancy is reduced to approximately 16 to 20 months (19). Due to the progressive motor impairments also affecting food and water intake, we sacrificed the mice at age 16 months on humanitarian grounds. By this time point, the casualties of the different experimental groups were recorded. Out of 34 ASA-expressing control mice, one FAAH-positive and one FAAH-negative mouse died at age 15 and 14 months, respectively (not shown). All other control mice reached an age of 16 months. Likewise, only two out of 13 MLD mice (expressing FAAH) died close to the end of the observation period of 16 months (Fig. 5*D*). FAAH deficiency, however, increased mortality of MLD mice significantly and ten of 20 mice died between ages 8 and 16 months. Thus, lack of FAAH diminishes the reduced life expectancy of MLD mice further. None of the mice developed hindlimb paralysis in the observation period.

### Behavioral performance of FAAH-deficient MLD mice

**Figure 6:**
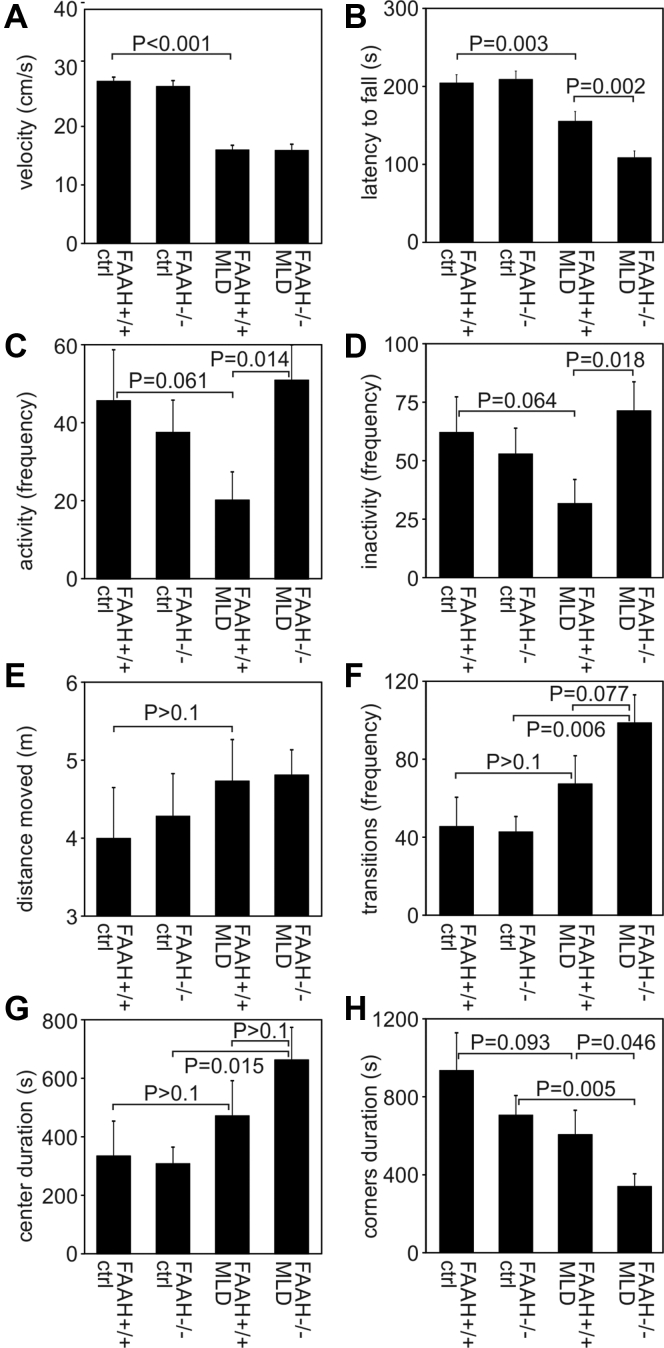
**Behavioral tests.** Bars represent means ± SEM of n = 13 to 16 mice per group. The indicated groups of 12-month-old mice were compared by Student's *t* test and the *p*-values are indicated. *A*, swimming velocity. For each mouse the average speed was calculated from the three consecutive trials. *B*, rotarod performance. The statistics includes the individual times mice were able to stay on the accelerating rotarod (latency to fall) in the four consecutive trials. *C*, frequency of activity. Activity used as a measure for mobility is defined as a movement that causes a total pixel change of the recorded body shape above a certain threshold. *D*, frequency of inactivity state. Is different from (*C*) due to another threshold value used to define inactivity. *E*, distance moved. Total length of the path covered by each mouse within 30 min. *F*, frequency of zone transitions. This is the number of times a mouse moves from one of three defined zones (center, border, corner) to another zone. *G*, center duration. Cumulative time that the mouse spent in the center zone of the arena. *H*, corner duration. Cumulative time spent in the four corner zones of the arena.

As shown previously, MLD mice develop progressive emotional deficits, hyperactivity, and coordination impairments (20). To evaluate possible effects of FAAH deficiency on these parameters, we used tests appropriate to measure the severity of behavioral alterations typical for MLD mice. For this purpose, we compared swimming velocity, rotarod performance, and open field behavior of (i) wild-type mice (FAAH+/+ ctrl), (ii) FAAH-knockout mice (FAAH−/− ctrl), (iii) MLD mice (FAAH+/+, MLD) and (iv) FAAH-deficient MLD mice (FAAH−/− MLD). All mice were 12 months of age. Importantly, FAAH deficiency alone had no detectable effect on the performance in any of the tests applied (compare the first two bars in the histograms of Fig. 6, *A*–*H*). As expected, significant behavioral changes were seen, however, in MLD mice compared with wild-type controls (compare the first with the third bar). Thus, the swimming velocity of MLD mice was severely reduced (Fig. 6*A*) and rotarod tests revealed deficits in motor coordination and balance (Fig. 6*B*). Furthermore, open field tests revealed trends for less frequent changes between phases of activity and inactivity (Fig. 6, *C* and *D*) with a simultaneous increase of migration distances (Fig. 6*E*), more frequent transitions between center and border zones of the cage (Fig. 6*F*), and a trend to spent less time in the corners and more in the center (Fig. 6, *G* and *H*). These alterations in the open field behavior were not significant, but in accordance with the previously detected increased anxiety-related exploratory activity of MLD mice (20).

Important conclusions can be drawn from the comparison of FAAH-expressing MLD mice and FAAH-deficient MLD mice (compare the last two bars in the histograms of Fig. 6, *A*–*H*). Whereas FAAH deficiency has no impact on the low swimming velocity of MLD mice (Fig. 6*A*), it further worsened the weak rotarod performance and the time it took the mouse to fall off the rod was reduced by an additional ∼30% (Fig. 6*B*). Additional effects of FAAH deficiency became apparent also in the open fields tests. Here, FAAH-deficient MLD mice switched ∼2.5-fold more often between phases of activity and inactivity, which led to a formal normalization of this parameter (Fig. 6, *C* and *D*). Notably, the shift to shorter walks and shorter breaks did not affect the total distances covered by the mice within the observation period (Fig. 6*E*). It did, however, correlate with a tendency for an increased mobility between the different zones of the cage (Fig. 6*F*) and the time spent in the corners was further reduced (Fig. 6, *G* and *H*).

### Anandamide and cytokine levels of FAAH-deficient MLD mice

**Figure 7:**
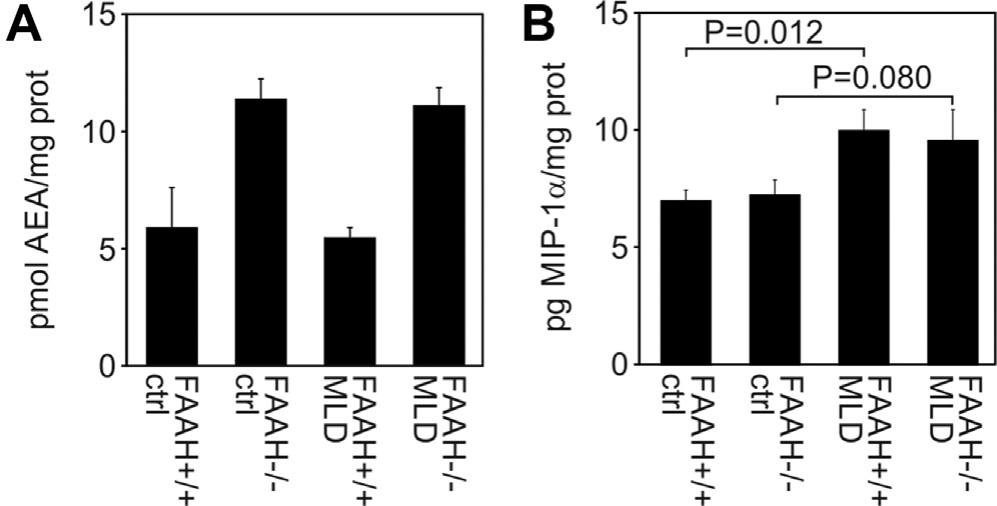
**Anandamide and cytokine levels of total brain.***A*, anandamide levels of the four indicated groups of mice at age 12 months. Bars represent means ± SEM. The size of the two groups of MLD mice was n = 5. Control groups comprised only n = 2 animals so that a statistical evaluation was not possible. *B*, levels of MIP-1α. Bars represent means ± SEM of n = 5 mice per group (MLD-mice) and three mice per group (non-MLD mice), respectively. Groups were compared by Student's *t* test and the *p*-values are indicated.

FAAH is the principal enzyme that degrades the neurotransmitter anandamide (AEA) (21). To evaluate the consequences of FAAH deficiency on the levels of this endocannabinoid, we quantified AEA in total brains of the four cohorts of mice by mass spectrometry. This analysis revealed a significant twofold increase in both groups of FAAH-deficient mice (Fig. 7*A*). Thus, FAAH deficiency increased AEA concentrations and ASA deficiency had no additional effect on the elevated levels.

To detect possible effects of FAAH deficiency on neuroinflammation typical of MLD-mice approaching 2 years of age (22), we evaluated the cytokine profiles of the four mouse cohorts at age 16 months. In this investigation we included macrophage inflammatory protein-1α (MIP-1α), the first cytokine that is upregulated in the disease progression of MLD mice, five cytokines being elevated at end-stage disease [MIP-1β, monocyte chemotactic protein-1 (MCP-1), RANTES, interleukin-1β, interleukin-6], and the two classical proinflammatory cytokines tumor necrosis factor-α (TNF-α) and interferon-γ (IFN-γ) both of which remain unchanged in MLD mice (22). The measurements reproduced part of our previous findings by showing significant upregulation of the lead cytokine MIP-1α in MLD mice compared with wild-type controls (Fig. 7*B*). Importantly, the absolute levels of this chemokine were indistinguishable between FAAH-expressing and FAAH-deficient MLD mice. Also for the other seven cytokines identical levels were measured in FAAH-expressing and FAAH-deficient MLD mice (not shown). In contrast to MIP-1α, none of them was significantly upregulated in any of the two groups of MLD mice compared with non-MLD controls. Thus, FAAH deficiency did not enhance the early stage of neuroinflammation seen in the present study.

## Discussion

Loading assays, based on the addition of radioactively labeled sulfatide to the medium of cultured patient cells, is a sensitive method to evaluate the metabolism of sulfatide and a classical means to diagnose MLD. To avoid radiolabeled sulfatide being expensive and increasingly difficult to obtain, we used fluorescently labeled NBD sulfatide for cell loading. After separation on TLC plates the limit of detection was as low as 10 fmol per band and thus lower than for ^3^H- or ^14^C-labeled sulfatide (data not shown). To further elevate the sensitivity of the assay, we increased the cellular uptake of NBD sulfatide by preincubation with FuGENE HD, a cationic cell transfection reagent. Cellular uptake was increased by a factor of 3 to 4 supposedly due to the incorporation of the anionic sulfatide into complexes with positive surface charges that can bind to the negatively charged cell surfaces. The high sensitivity of our nonradioactive loading assay allowed us to identify an ASA-independent sulfatide-hydrolyzing activity of macrophages and microglial cells. This activity was demonstrated by the release of the fatty acid from sulfatide in the complete absence of lysosomal ASA activity. No such reaction was detectable in ASA-deficient astrocytoma cells. Of note, more than 25 years ago evidence for an ASA-independent pathway of sulfatide degradation has already been provided for immortalized B-cells from a late infantile MLD patient (10). Furthermore, a nonlysosomal enzyme hydrolyzing sulfatide has been identified in wild-type mouse brain about the same time (11). In both cases, the responsible enzyme(s) could, however, not be determined.

**Figure 8:**
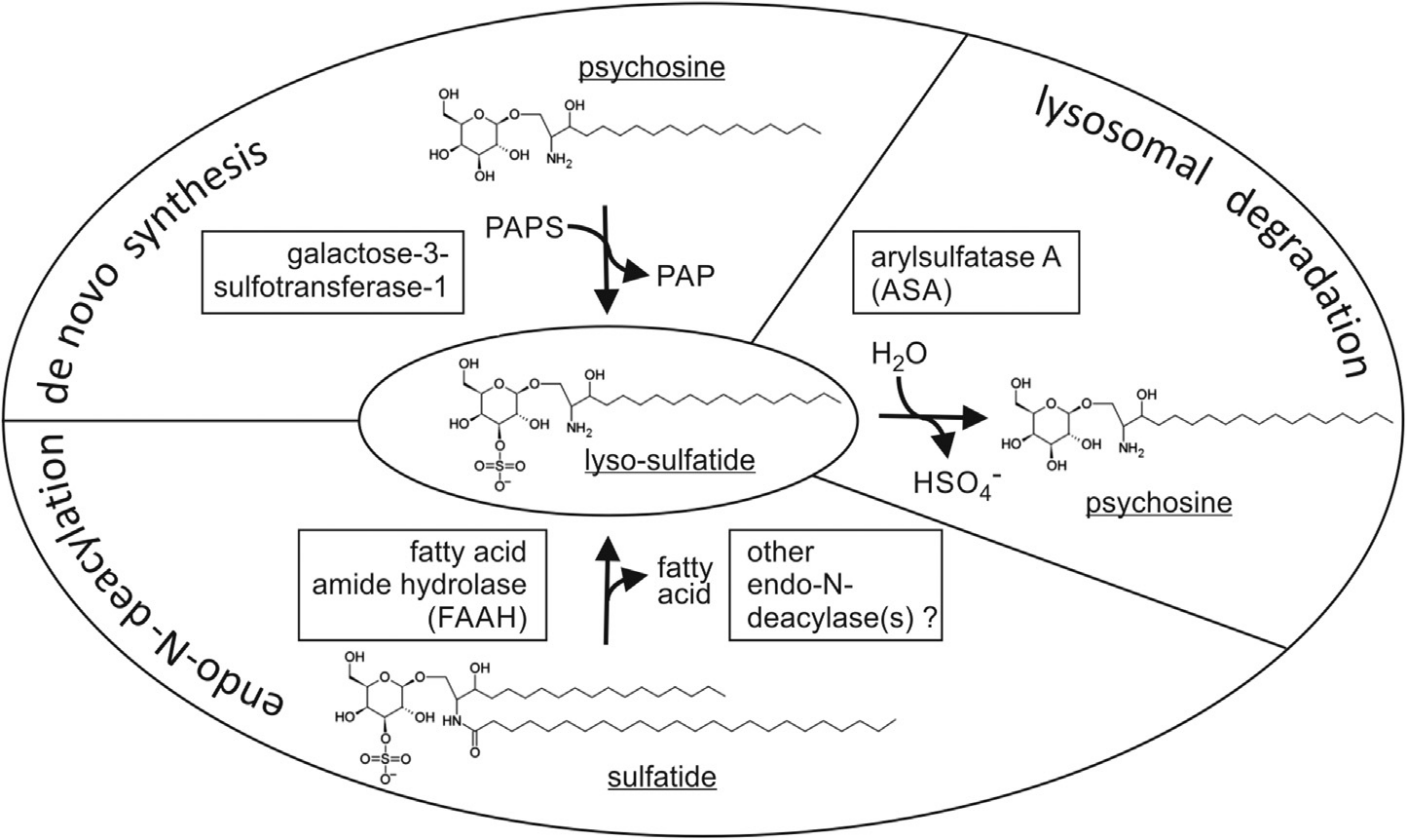
**Model of the lyso-sulfatide metabolism combining established and hypothetical pathways.** Sulfation of psychosine by the galactose-3-sulfotransferase-1 has been proposed by Lin and Horowitz (9). ASA converts lyso-sulfatide to psychosine (45). For further details, see main text.

To identify the putative endo-N-deacylase of macrophages and microglial cells, we tested 13 amidases that, based on published enzyme data, might fulfill the requirements of such a reaction. In an expression cloning approach, the enzymes were individually overexpressed in ASA-deficient astrocytoma cells and their potential to liberate the fatty acid from sulfatide was measured by HPLC. Among the enzymes tested, only FAAH increased the release of fatty acid from sulfatide. ASAH1 has been implicated in the deacylation of the GSLs glucosylceramide, galactosylceramide, and globotriaosylceramide to their corresponding lyso-forms (8). It is, therefore, important to point out that no deacylation of sulfatide was detectable after transient or stable overexpression of ASAH1. An *in vivo* function of FAAH in the metabolism of (unmodified) sulfatide was verified by crossing ASA knockout mice with FAAH knockout mice with the resulting double-deficient mice showing significantly reduced levels of lyso-sulfatide. Still, sizable ∼80% of the lyso-sulfatide level seen in FAAH-expressing MLD mice persisted, indicating that FAAH-independent pathways contribute to the bulk of lyso-sulfatide production. Additional experiments are required to find out if other sulfatide-hydrolyzing endo-N-deacylases exist and/or if *de novo* synthesis by sulfation of psychosine *via* the galactose-3-sulfotransferase-1 plays a major role in lyso-sulfatide formation (9) (Fig. 8).

FAAH is a component of the endocannabinoid system comprising endocannabinoids acting as retrograde neurotransmitters, cannabinoid receptors signaling through G-proteins and hydrolases terminating the action of released endocannabinoids by enzymatic cleavage (21). FAAH hydrolyzes the endocannabinoid AEA (N-arachidonoylethanolamine) to arachidonic acid and ethanolamine. The enzyme is, however, quite promiscuous and accepts substrates of varying fatty acid chain length, degree of saturation, and head group structure (23). Although sulfatide might fall into the broad spectrum of FAAH substrates, it has not been identified as such to date. FAAH is present in the brain, liver, kidney, and many other tissues. Also, primary macrophages and microglia express FAAH (24, 25, 26, 27). At first glance, a role of FAAH in the catabolism of sulfatide is surprising as FAAH is not lysosomal but an integral membrane protein of the ER. Sulfatide is synthesized from galactosylceramide in the Golgi apparatus (28), and a direct encounter of FAAH with sulfatide, therefore, seems impossible. Lysosomal storage might, however, result in a translocation of accumulating sulfatide to other cell compartments, especially to the endoplasmic reticulum, as previously suggested for glucosylceramide in the lysosomal storage disease Gaucher disease (29). Such a retrograde transfer from the lysosome to the ER might be accomplished by cytosolic lipid transporters (1) or interorganelle membrane contact sites (12). The crystal structure of FAAH reveals a putative substrate entry channel that is directly accessible for lipids of the ER membrane (30). This might allow access of sulfatide to the active site of the enzyme without the need of prior extraction from the membrane *via* activator proteins (saposins) that are indispensable for lysosomal degradation (1, 2).

It is striking that FAAH shares features with the aforementioned nonlysosomal sulfatide-hydrolyzing enzyme that has been partially purified from mouse brain (11). Thus, the molecular mass and pI of the latter were reported to be 66 kDa and 7.7, respectively, compared with 67 kDa and 7.8 determined for FAAH (21). Furthermore, both enzymes are nonglycosylated and membrane-bound. It is tempting to speculate that we have reidentified the same enzyme and pathway that has been partially uncovered by a classical biochemical approach more than 2 decades ago. It is even conceivable that the nonlysosomal sulfatide-hydrolyzing enzyme of immortalized human B-cells was *de facto* FAAH (10). The enzyme neither accepted lyso-sulfatide nor p-nitrocatecholsulfate as a substrate, which, as the authors mentioned, is remarkable for a sulfatase that accepts sulfatide. The substrate specificity would be, however, logical for an endo-N-deacylase as both substrates are devoid of an N-linked fatty acid.

Another important finding of the present study is that genetic elimination of FAAH activity, albeit lowering the lyso-sulfatide level in the CNS, exacerbated the disease phenotype of ASA-deficient MLD mice. Thus, double-deficient mice showed a reduced rotarod performance, increased anxiety-related exploratory activity, aggravated peripheral neuropathy, and reduced life span compared with conventional MLD mice. The more severe phenotype in the presence of reduced lyso-sulfatide levels cannot be explained by the common pathophysiological model of glycosphingolipidoses. This model claims that the lyso-forms of the accumulating GSLs are cytotoxic and promote the progression of disease by disturbing signal transduction pathways, energy homeostasis, and other cellular processes (4). Aggravation of the disease phenotype is also difficult to explain in view of the known anti-inflammatory and neuroprotective effects exerted by inhibition of FAAH activity in many animal models of neurological diseases (31). Only recently, it has been shown, for example, that oral treatment of sphingomyelinase-deficient mice (model of the lysosomal storage disease Niemann–Pick type A/B) with FAAH inhibitors alleviated neuroinflammation, retarded neurodegeneration, improved behavioral alterations, and extended life span (32). To find a possible explanation for the severe phenotype of FAAH-deficient MLD mice, we quantified AEA and cytokines in total brain. The measurement of cytokines did, however, not reveal any evidence that FAAH deficiency enhances the neuroinflammatory process typical of aged MLD mice (22). Likewise, ASA deficiency has no additional effect on the increased AEA level of FAAH-deficient mice. Thus, there is no obvious explanation for the more severe phenotype of double-deficient mice to date. Cytotoxic effects of AEA have been described (33, 34), and it might be speculated that certain brain cell types are particularly vulnerable to AEA-induced apoptosis if they are predamaged by sulfatide or lyso-sulfatide storage. Alternatively, FAAH deficiency might cause the formation of a lipid species with higher cytotoxicity than lyso-sulfatide. Shotgun lipidomics or LC-MS analysis of lipids would be most straightforward to identify such a hypothetical compound. Clearly it would be of interest to evaluate why a reduction of the FAAH activity has deleterious effects in MLD mice, but ameliorates the disease phenotype of mouse models of other CNS diseases (31). It would also be interesting to analyze if supraphysiological FAAH activities have the opposite effect and mitigate the disease progression in MLD mice. Because allosteric activators of FAAH are not known, this question has to be addressed by transgenic overexpression of FAAH in the MLD background.

## Experimental procedures

### Cells

Two immortalized astrocytoma cell lines, wild-type 11+/+C1 cells and ASA-deficient 17−/−A1 cells, were used (13). Unless otherwise indicated they were cultured in Dulbecco's modified Eagle's medium (DMEM) without phenol red (Thermo Fisher Scientific, cat. no. 31053044). ASA-deficient mixed brain cell cultures and microglial cells were isolated from the brains of newborn ASA knockout mice (35). ASA-deficient macrophages were prepared from bone marrow of adult ASA knockout mice (36) using Fischer's medium supplemented with 10% fetal bovine serum, 100 U/ml penicillin, 100 μg/ml streptomycin (all from Thermo Fisher Scientific), and 50 ng/ml macrophage colony stimulating factor (Peprotech).

### cDNAs and cloning of expression plasmids

E.coli stab cultures were purchased from Invitrogen and ImaGenes-SourceBioscience, respectively (Table S1). Plasmid DNA was prepared by standard methods and the cDNA inserts were sequenced in both directions (Eurofins Genomics). Sequence-verified full-length cDNAs were PCR-cloned into the eukaryotic expression plasmid pcDNA3 (Thermo Fisher Scientific). A double-stranded DNA sequence encoding a C-terminal myc-tag preceded by a Ser-Gly_4_-Ser linker was synthesized by annealing of two complementary oligonucleotides (Eurofins Genomics) and cloned in frame to generate myc-tagged proteins. Exceptions were ASAH1, NAAA, and FAAH, which were expressed as untagged proteins to avoid interference with proteolytic processing and/or hydrolytic activity suggested by published data. The plasmid pcDNA3-ASA-myc harboring the myc-tagged full-length human ASA cDNA was cloned accordingly. The transmembrane domain of the exoenzyme DPEP1 was deleted to obtain a soluble enzyme variant. All cDNAs were of human origin except NAAA, for which only a murine full-length clone was available.

### Transfection and western blotting

17−/−A1 astrocytoma cells were transiently transfected with Turbofect (Thermo Fisher Scientific) following the instructions of the manufacturer. To generate stably transfected cells, 500 μg/ml G418 disulfate (VWR Chemicals) was added to the medium for 10 to 14 days and growing single clones were isolated by aspiration with a pasteur pipette. Transgene expression of transfected cells was verified by immunoblotting using anti-myc antibodies and, for ASAH1, NAAA, and FAAH, protein-specific antibodies, respectively. The following primary antibodies and dilutions were used: (i) mouse monoclonal α-c-myc antibody 9e10 (Santa Cruz; sc-40; 1:5000); (ii) goat polyclonal α-FAAH V-17 antiserum (Santa Cruz, sc-26427, 1:1000); (iii) goat polyclonal α-ASAHL (NAAA) G-13 antiserum (Santa Cruz, sc-104081, 1:200); (iv) rabbit polyclonal α-ASAH1 antiserum (kindly provided by K. Sandhoff, Bonn, Germany; 1:1000). The following secondary antibodies were used: (i) peroxidase-conjugated bovine α-goat antibodies (Dianova; 805-035-180; 1:10,000); (ii) peroxidase-conjugated goat α-rabbit antibodies (Dianova, 111-035-003; 1:10,000).

### Sulfatide loading and lipid extraction

The GSL metabolism of cultured cells was analyzed using the sulfatide fluorescent analogue N-[12-[(7-nitro-2-1,3-benzoxadiazol-4-yl)amino]dodecanoyl]-cerebroside 3-sulfate (NBD-sulfatide; Matreya). In this compound the NBD fluorophore is linked to the terminal (omega) carbon atom of the N-linked fatty acid. In general, 5 nmol NBD sulfatide was added to subconfluent cells in a 35 mm dish. For *translipofection*, 5 nmol NBD sulfatide was dissolved in 1.5 μl methanol and mixed with 1.5 μl FuGENE HD transfection reagent (Promega) prior to loading. In pretests also TurboFect, ExGen 500, and Lipofectamine 2000 transfection reagent were used instead of FuGENE (all from Thermo Fisher Scientific). Loading by complexation with bovine serum albumin (BSA) and synthetic low-density lipoproteins (sLDL) was as described (37, 38). After incubation for 6 h at 37 °C, cells were washed and cultured with fresh medium for 18 h. Then, total lipid was isolated from cells and/or conditioned medium by solid-phase extraction using self-made RP-18 LiChroprep (Merck) columns with 2 ml bed volume prewashed once with 1 ml methanol and twice with 1 ml H_2_O bidest each. After loading the samples, the flow-through was discarded and the columns were washed four times with 1 ml each of 4 mM hydrochloric acid. Lipids were eluted with 6 ml chloroform/methanol 1:1 (v/v) and dried under a nitrogen stream. The dry lipids were dissolved in 200 μl chloroform/methanol 1:1 (v/v) for separation by thin-layer chromatography (TLC) and in 250 μl methanol/H_2_O bidest/85% phosphoric acid 550:450:1.5 (v/v/v) for separation by HPLC, respectively.

### Lipid analysis

For TLC, appropriate volumes of lipid extracts were applied to high-performance TLC plates (Merck) with an automatic TLC sampler 4 (CAMAG). Separation was with chloroform/methanol/acetic acid 190:9:1 (v/v/v) as solvent system. NBD sulfatide (Matreya), NBD galactosylceramide (Avanti Polar Lipids), and NBD dodecanoic acid (Invitrogen-Thermo Fisher Scientific) were coseparated as standards. Metabolic products harboring an NBD-labeled fatty acid were identified with a FLA-5100 fluorescence scanner (Fujifilm Life Science) at an excitation and emission wave length of 473 and 537 nm, respectively. For HPLC, a Bio-Tek HPLC system (Kontron AG) equipped with a Zorbax C18-column (2.1 mm × 150 mm; Agilent Technologies) and an FP-2020 fluorescence detector (Jasco) was used. The wavelength for excitation was 465 nm and for emission 535 nm. Mobile phases consisted of methanol/H_2_O bidest/85% phosphoric acid in different volume fractions: (A) 550:450:1.5, (B) 750:250:1.5, (C) 1000:0:1.5. For the separation of lipids, the column was run with a constant flow rate of 0.5 ml/min at room temperature. The gradient cycle was 50 min in length with a 5 min isocratic segment with solvent (A), a 15 min linear gradient to solvent (B), a 15 min isocratic segment with solvent (C), and a 15 min isocratic segment with solvent (A). Lyso-sulfatide and sulfatide levels of mouse brain were quantified by mass spectrometry on a Xevo TO-S triple quadrupole mass spectrometer (Waters GmbH) (39) and TLC, respectively (40).

### Anandamide analysis

AEA levels of total brain were analyzed by LC–mass spectrometry (MS) essentially as described (41) using deuterated AEA (AEA-d8) as an internal standard (Cayman Chemical). Following extraction with acetonitrile (ACN), AEA chromatography was performed on a Dionex Ultimate 3000 RSLC nano HPLC system (Dionex GmbH). The autosampler was operated in μl-pickup mode. Dried extracts were dissolved in 10 μl 95% ACN, 0.1% formic acid (FA). Solvent A was 0.1% FA. Two microliter was injected onto a C18 analytical column (self-packed 300 mm length, 75 μm inner diameter, ReproSil-Pur 120 C18-AQ, 3 μm, Dr Maisch GmbH) equilibrated with 35% solvent B (90% ACN, 0.1% FA). AEA was separated during a linear gradient from 35% to 98% solvent B at 400 nl/min. The nanoHPLC was coupled online to an Orbitrap Fusion Lumos mass spectrometer (Thermo Fisher Scientific). Ions between 200 and 600 m/z were scanned in the Orbitrap detector every 3 s with a resolution of 60,000 at m/z = 200 (maximum fill time 50 ms, AGC target 400,000). In a parallel reaction monitoring method, selected ions ([d8-AEA+H]+, 356.3399 Da; [d8-AEA+Na]+, 378.3219 Da; [AEA+H]+, 348.2897 Da; [AEA+Na]+, 370.2717 Da) were subjected to higher-energy CID (HCD, 0.7 Da quadrupole isolation, threshold, normalized energy 30%) and fragments analyzed in the Orbitrap analyzer (resolution 7500, maximum fill time 22 ms, target 50,000). Data analysis was done with Skyline (42) software using areas of extracted ion chromatograms of two most abundant precursor isotope masses and a product ion: 62.0600 Da or 62.0663 Da for endogenous or d8-AEA respectively. Typical mass deviation was <2 ppm (MS1) and <15 ppm (MS2). The abundance ratio between AEA and d8-AEA was used for determination of AEA quantities. Technical replicates with highest signal-to-noise ratios of internal standard were used.

### Cytokine analysis

Brains were dissected, cut midsagitally, and one hemisphere (about 200 mg wet weight) was homogenized in 2 ml ice-cold phosphate-buffered saline (PBS) pH 7.4 supplemented with Halt protease inhibitor cocktail (Thermo Fisher Scientific) and 5 mM EDTA using an Ultra-Turrax T25 homogenizer (Janke & Kunkel). Homogenates were centrifuged for 5 min at 10,000*g* and 4 °C. Protein concentrations of the supernatants were determined using the Bio-Rad Dc assay (Bio-Rad). Cytokine levels were measured with ABTS-ELISA kits according to the manufacturer's recommendations (Peprotech). For the ELISAs appropriate volumes of the supernatant supplemented with 0.05% Tween-20 were used. Homogenates of 3 to 5 mice per group and eight dilutions of each cytokine standard were measured in duplicates. The resulting calibration curves were used to determine the cytokine levels that were subsequently normalized on the corresponding protein concentration of the homogenate.

### Life-cell fluorescence microscopy

Cells were loaded with the Golgi marker Invitrogen BODIPY TR ceramide (Thermo Fisher Scientific) for 30 min according to the recommendations of the manufacturer. Then, the cells were washed and BODIPY 500/510-labeled sulfatide was loaded by *translipofection* as described before. The labeled sulfatide was synthesized by standard methods from lyso-sulfatide and BODIPY 500/510 C12-fatty acid (Thermo Fisher Scientific) (14). Fluorescence was monitored in an Axiovert 200M inverted microscope equipped with appropriate fluorescence filters, an AxioCam MRc camera in monochrome mode and the AxioVision 4.8.2. software (all from Carl Zeiss).

### Enzymes and cell-free *in vitro* assays

Recombinant human FAAH expressed in Sf21 insect cells was from Cayman Chemicals. The activity of FAAH was measured with 7-amino-4-methyl coumarin arachidonoyl amide (AMCAA, Cayman Chemicals) that is cleaved to arachidonic acid and highly fluorescent 7-amino-4-methyl coumarin (43). To test FAAH-catalyzed hydrolysis of sulfatide, NBD sulfatide (1 nmol) was dried in a stream of nitrogen, dissolved in 2 μl methanol, and mixed with 200 μl FAAH reaction buffer (125 mM Tris-HCl pH 9.0, 1 mM EDTA, 4.7 mM sodium taurodeoxycholate) by ultrasonication. After adding four units of recombinant human FAAH, the mixture was incubated for 24 h at 37 °C. The reaction was stopped and lipids were extracted with 1 ml chloroform/methanol 1:1 (v/v). Following vigorous vortexing and phase separation by centrifugation, the organic phase was collected and dried under a stream of nitrogen. The residue was dissolved in 200 μl chloroform/methanol 1:1 (v/v) and analyzed by TLC. As a negative control, the competitive FAAH-inhibitor URB597 (Cayman Chemicals) was added at a final concentration of 1 μM. As a positive control for the deacylation reaction under investigation, bacterial sphingolipid ceramide N-deacylase (SCDase; Takara) was used in FAAH reaction buffer without sodium taurodeoxycholate. SCDase efficiently hydrolyzes the N-acyl linkage between the fatty acid and sphingosine base of GSLs, including NBD sulfatide (see Fig. 4, *E* and *G*).

### Mice

Mice with a deficiency of murine ASA (mASA−/−) being double transgenic (tg) for cerebroside sulfotransferase (PLP-CST) and the inactive human ASA (hASA) mutant hASAc69s represent an aggravated mouse model of MLD (19). For reasons of simplicity, they are here briefly designated as MLD-mice. They were crossed with FAAH knockout mice (FAAH−/−; kindly provided by Andreas Zimmer, Bonn, Germany) to generate four genotype combinations: (i) FAAH−/− mASA−/− hASAc69s tg PLP-CST tg (FAAH-deficient MLD-mice); (ii) FAAH+/+ mASA−/− hASAc69s tg PLP-CST tg (FAAH-expressing MLD-mice); (iii) FAAH−/− mASA ± hASAc69s tg PLP-CST tg (FAAH-deficient control mice); (iv) FAAH+/+ mASA ± hASAc69s tg PLP-CST tg (FAAH-expressing control mice). Thus, all four groups were double transgenic for CST and hASAc69s and differed by the combination of FAAH and mASA knockout alleles. Animals were maintained on a 12-h-light and 12-h-dark schedule with *ad libitum* access to food and water. Mice were kept according to the German “Tierschutzgesetz” and the European Union directive 2010/63/EU on the protection of animals used for scientific purposes. Breeding, handling, and investigation of the mice were approved by the local authorities (permit no. 84-02.04.2016.A394; Landesamt für Natur, Umwelt und Verbraucherschutz). To evaluate the degree of hypertrophic neuropathy, sciatic nerves of full length were dissected from female mice (44), the two nerves per mouse were combined, and their wet weights were determined by weighing on an analytical balance.

### Behavioral tests

Mouse behavior was tested at an age of 12 months. All 63 mice analyzed (13–18 mice per group) were born in a small time interval of 16 days. They were trained and tested one by one in a fixed order on identical days. Motor coordination was evaluated using a rotarod apparatus (Ugo Basile, Model 47600) at an accelerated speed mode with a ramp duration of 300 s and a minimum and maximum speed of 4 and 40 rpm/min, respectively. Mice were accustomed to the task on four consecutive days at a constant speed of 4 rpm for 2 min. On each of the following 4 days the mice were tested at the accelerating speed mode once. The time spent on the rotarod (latency to fall) was recorded. The swimming velocity was measured in a self-constructed opaque swimming pool with the dimensions 150 × 10 × 15 cm (length × width × height). The water temperature and depth were 26 °C and 10 cm, respectively. An accessible platform of 10 × 10 cm was placed at one end of the lane and allowed the mice to get out of the water. The mice were first trained on four consecutive days to swim increasing distances to the platform following a light gradient from bright to dark (one trial per day). On the following 3 days, mice were put into a 10 × 10 cm starting area opposite to the platform and filmed with a video camera from above. The time needed to cover the last 100 cm to the platform was extracted from the recordings and the swimming velocity (m/s) was calculated. Motor activity, anxiety-related responses, and exploratory activities were analyzed by open field tests using a dimly lit arena measuring 30 × 30 cm (30 cm high) divided into a wall zone (7 cm wide), corner zones (7 × 7 cm), and a center zone (16 × 16 cm). Mice were placed into the center of the arena and tracked for 30 min using a computer-operated EthoVision camera system and the EthoVision XT9 software (Noldus Information Technology). Various exploratory activities and anxiety-related responses were extracted from the tracks including the distance moved, the mean velocity, the duration and frequency of activity/inactivity intervals, and the times spent in the wall-, corner-, and center zones. Due to significant gender-specific differences and a higher variability of the behavior of male mice in the open field tests, only female mice were evaluated.

## Data availability

The authors confirm that the data supporting the findings of this study are available within the article, its supporting materials, and from the corresponding author, Ulrich Matzner (Institute of Biochemistry and Molecular Biology, Medical Faculty, University of Bonn, Email: matzner@uni-bonn.de) upon reasonable request, respectively.

## Supporting information

This article contains supporting information (17).

## Conflict of interest

The authors declare that they have no conflicts of interest with the contents of this article.

## References

[1] Schulze H., Sandhoff K. (2014). Sphingolipids and lysosomal pathologies. Biochim. Biophys. Acta.

[2] Breiden B., Sandhoff K. (2019). Lysosomal glycosphingolipid storage diseases. Annu. Rev. Biochem..

[3] von Figura K., Gieselmann V., Jaeken J., Scriver C.R., Beaudet A.L., Sly W.S., Valle D., Childs B., Kinzler K.W., Vogelstein B. (2001). The Metabolic and Molecular Bases of Inherited Disease.

[4] Suzuki K. (1998). Twenty five years of the “psychosine hypothesis”: A personal perspective of its history and present status. Neurochem. Res..

[5] Hannun Y.A., Bell R.M. (1987). Lysosphingolipids inhibit protein kinase C: Implications for the sphingolipidoses. Science.

[6] Toda K., Kobayashi T., Goto I., Kurokawa T., Ogomori K. (1989). Accumulation of lysosulfatide (sulfogalactosylsphingosine) in tissues of a boy with metachromatic leukodystrophy. Biochem. Biophys. Res. Commun..

[7] Hans M., Pusch A., Dai L., Racke K., Swandulla D., Gieselmann V., Kappler J. (2009). Lysosulfatide regulates the motility of a neural precursor cell line via calcium-mediated process collapse. Neurochem. Res..

[8] van Eijk M., Ferraz M.J., Boot R.G., Aerts J.M.F.G. (2020). Lyso-glycosphingolipids: Presence and consequences. Essays Biochem..

[9] Lin Y.N., Horowitz M.I. (1982). Cerebroside sulfotransferase in rat gastric mucosa. Int. J. Biochem..

[10] Tempesta M.C., Salvayre R., Levade T. (1994). Functional compartments of sulphatide metabolism in cultured living cells: Evidence for the involvement of a novel sulphatide-degrading pathway. Biochem. J..

[11] Sundaram S.K., Fan J.H., Lev M. (1995). A neutral galactocerebroside sulfate sulfatidase from mouse brain. J. Biol. Chem..

[12] Henne W.M. (2017). Discovery and roles of ER-endolysosomal contact sites in disease. Adv. Exp. Med. Biol..

[13] Habetha M. (2001). Sulfatide does not induce apoptosis in arylsulfatase A-deficient cells. Neurosci. Res. Commun..

[14] Schwarzmann G. (2018). Synthesis of fluorescent gangliosides. Methods Mol. Biol..

[15] Pohl A., Lage H., Müller P., Pomorski T., Herrmann A. (2002). Transport of phosphatidylserine via MDR1 (multidrug resistance 1) P-glycoprotein in a human gastric carcinoma cell line. Biochem. J..

[16] Polten A., Fluharty A.L., Fluharty C.B., Kappler J., von Figura K., Gieselmann V. (1991). Molecular basis of different forms of metachromatic leukodystrophy. N. Engl. J. Med..

[17] Lübke T., Lobel P., Sleat D.E. (2009). Proteomics of the lysosome. Biochim. Biophys. Acta.

[18] Furusato M., Sueyoshi N., Mitsutake S., Sakaguchi K., Kita K., Okino N., Ichinose S., Omori A., Ito M. (2002). Molecular cloning and characterization of sphingolipid ceramide N-deacylase from a marine bacterium, Shewanella alga G8. J. Biol. Chem..

[19] Ramakrishnan H., Hedayati K.K., Lüllmann-Rauch R., Wessig C., Fewou S.N., Maier H., Goebel H.H., Gieselmann V., Eckhardt M. (2007). Increasing sulfatide synthesis in myelin-forming cells of arylsulfatase A-deficient mice causes demyelination and neurological symptoms reminiscent of human metachromatic leukodystrophy. J. Neurosci..

[20] Matthes F., Stroobants S., Gerlach D., Wohlenberg C., Wessig C., Fogh J., Gieselmann V., Eckhardt M., D'Hooge R., Matzner U. (2012). Efficacy of enzyme replacement therapy in an aggravated mouse model of metachromatic leukodystrophy declines with age. Hum. Mol. Genet..

[21] Lu H.C., Mackie K. (2016). An introduction to the endogenous cannabinoid system. Biol. Psychiatry.

[22] Stein A., Stroobants S., Gieselmann V., D'Hooge R., Matzner U. (2015). Anti-inflammatory therapy with simvastatin improves neuroinflammation and CNS function in a mouse model of metachromatic leukodystrophy. Mol. Ther..

[23] Lang W., Qin C., Lin S., Khanolkar A.D., Goutopoulos A., Fan P., Abouzid K., Meng Z., Biegel D., Makriyannis A. (1999). Substrate specificity and stereoselectivity of rat brain microsomal anandamide amidohydrolase. J. Med. Chem..

[24] Maccarrone M., van der Stelt M., Rossi A., Veldink G.A., Vliegenthart J.F., Agrò A.F. (1998). Anandamide hydrolysis by human cells in culture and brain. J. Biol. Chem..

[25] Di Marzo V., Bisogno T., De Petrocellis L., Melck D., Orlando P., Wagner J.A., Kunos G. (1999). Biosynthesis and inactivation of the endocannabinoid 2-arachidonoylglycerol in circulating and tumoral macrophages. Eur. J. Biochem..

[26] Tanaka M., Yagyu K., Sackett S., Zhang Y. (2019). Anti-Inflammatory effects by pharmacological inhibition or knockdown of fatty acid amide hydrolase in BV2 microglial cells. Cells.

[27] Stella N. (2009). Endocannabinoid signaling in microglial cells. Neuropharmacology.

[28] Yaghootfam A., Sorkalla T., Häberlein H., Gieselmann V., Kappler J., Eckhardt M. (2007). Cerebroside sulfotransferase forms homodimers in living cells. Biochemistry.

[29] Elleder M. (2006). Glucosylceramide transfer from lysosomes--the missing link in molecular pathology of glucosylceramidase deficiency: A hypothesis based on existing data. J. Inherit. Metab. Dis..

[30] McKinney M.K., Cravatt B.F. (2005). Structure and function of fatty acid amide hydrolase. Annu. Rev. Biochem..

[31] Ren S.Y., Wang Z.Z., Zhang Y., Chen N.H. (2020). Potential application of endocannabinoid system agents in neuropsychiatric and neurodegenerative diseases-focusing on FAAH/MAGL inhibitors. Acta Pharmacol. Sin..

[32] Bartoll A., Toledano-Zaragoza A., Casas J., Guzmán M., Schuchman E.H., Ledesma M.D. (2020). Inhibition of fatty acid amide hydrolase prevents pathology in neurovisceral acid sphingomyelinase deficiency by rescuing defective endocannabinoid signaling. EMBO Mol. Med..

[33] Schwarz H., Blanco F.J., Lotz M. (1994). Anandamide, an endogenous cannabinoid receptor agonist inhibits lymphocyte proliferation and induces apoptosis. J. Neuroimmunol..

[34] Maccarrone M., Lorenzon T., Bari M., Melino G., Finazzi-Agro A. (2000). Anandamide induces apoptosis in human cells via vanilloid receptors. Evidence for a protective role of cannabinoid receptors. J. Biol. Chem..

[35] Kaminski D., Yaghootfam C., Matthes F., Reßing A., Gieselmann V., Matzner U. (2021). Brain cell type-specific endocytosis of arylsulfatase A identifies limitations of enzyme-based therapies formetachromatic leukodystrophy. Hum. Mol. Genet..

[36] Zhang X., Goncalves R., Mosser D.M. (2008). The isolation and characterization of murine macrophages. Curr. Protoc. Immunol..

[37] Monti E., Preti A., Novati A., Aleo M.F., Clemente M.L., Marchesini S. (1992). Uptake and metabolism of a fluorescent sulfatide analogue in cultured skin fibroblasts. Biochim. Biophys. Acta.

[38] Baillie G., Owens M.D., Halbert G.W. (2002). A synthetic low density lipoprotein particle capable of supporting U937 proliferation *in vitro*. J. Lipid Res..

[39] Mirzaian M., Kramer G., Poorthuis B.J. (2015). Quantification of sulfatides and lysosulfatides in tissues and body fluids by liquid chromatography-tandem mass spectrometry. J. Lipid Res..

[40] Matzner U., Herbst E., Hedayati K.K., Lüllmann-Rauch R., Wessig C., Schröder S., Eistrup C., Möller C., Fogh J., Gieselmann V. (2005). Enzyme replacement improves nervous system pathology and function in a mouse model for metachromatic leukodystrophy. Hum. Mol. Genet..

[41] Chen J., Paudel K.S., Derbenev A.V., Smith B.N., Stinchcomb A.L. (2009). Simultaneous quantification of anandamide and other endocannabinoids in dorsal vagal complex of rat brainstem by LC-MS. Chromatographia.

[42] Pino L.K., Searle B.C., Bollinger J.G., Nunn B., MacLean B., MacCoss M.J. (2020). The Skyline ecosystem: Informatics for quantitative mass spectrometry proteomics. Mass Spectrom. Rev..

[43] Ramarao M.K., Murphy E.A., Shen M.W., Wang Y., Bushell K.N., Huang N., Pan N., Williams C., Clark J.D. (2005). A fluorescence-based assay for fatty acid amide hydrolase compatible with high-throughput screening. Anal. Biochem..

[44] Bala U., Tan K.L., Ling K.H., Cheah P.S. (2014). Harvesting the maximum length of sciatic nerve from adult mice: A step-by-step approach. BMC Res. Notes.

[45] Breiden B., Sandhoff K. (2019). Emerging mechanisms of drug-induced phospholipidosis. Biol. Chem..

