# Supplementary material for: Deletion of fatty acid amide hydrolase reduces lyso-sulfatide levels but exacerbates metachromatic leukodystrophy in mice
Source: J Biol Chem. 2021 Aug 8;297(3):101064. doi: 10.1016/j.jbc.2021.101064 (PMC8435702; doi:10.1016/j.jbc.2021.101064)
Supplement: Supplemental Figure S1 [file mmc1.docx]

**(A)**

| **Score** | **Expect** | **Method** | **Identities** | **Positives** | **Gaps** |
| --- | --- | --- | --- | --- | --- |
| 32.0 bits(71) | 6e-05 | Compositional matrix adjust. | 33/137(24%) | 56/137(40%) | 13/137(9%) |

SCDase 516 HGLSEKGVYLVNRMIDMGMLIELDHMSAQTATSVMDIVEQRQYGGVITSHS--WMTDGTQ 573

GLS G +V + +G+LI+L H+S T + + Q VI SHS + ++

DPEP1 190 QGLSPFGQRVVKELNRLGVLIDLAHVSVATMKATL----QLSRAPVIFSHSSAYSVCASR 245

SCDase 574 GRLHPNTLRLAKVGGFMAPYNSNANHLGGS-------IDRYLQLIADTPFLPGVGLGTDM 626

+ + LRL K + N N++ + + +L I + VG G D

DPEP1 246 RNVPDDVLRLVKQTDSLVMVNFYNNYISCTNKANLSQVADHLDHIKEVAGARAVGFGGDF 305

SCDase 627 SGLGAQAGPRDDAATNP 643

G+ +D + P

DPEP1 306 DGVPRVPEGLEDVSKYP 322

**(B)**

| **Score** | **Expect** | **Method** | **Identities** | **Positives** | **Gaps** |
| --- | --- | --- | --- | --- | --- |
| 26.2 bits(56) | 0.001 | Compositional matrix adjust. | 24/71(34%) | 32/71(45%) | 5/71(7%) |

SCDase 119 DDYMKLANPQASLVSPFVVRLHPYSRVKTQGWNIVYIPYCTGDLYAGDKVAVYDDPSGKK 178

+D +K A VS V HP + G + Y P CT ++ G V Y D +GK+

CTSS 235 EDVLKEAVANKGPVSVGVDARHPSFFLYRSG--VYYEPSCTQNVNHGVLVVGYGDLNGKE 292

SCDase 179 PPLV---WHHN 186

LV W HN

CTSS 293 YWLVKNSWGHN 303

**Supporting Fig. 1.** Sequence alignments of non-human sphingolipid ceramide N-deacylases (SCDases) with non-redundant protein sequences (nr) from Homo sapiens (taxid: 9606) using BlastP (https://blast.ncbi.nlm.nih.gov/Blast.cgi). **(A)** The putative active site of the SCDase from Shiwanella algae (amino acids 516-643) (18) shares 24% sequence identity with human dipeptidase 1 (DPEP1; NCBI reference sequence: NP_001121613.1). **(B)** SCDase from Pseudomonas sp. TK-4 (amino acids 119-186; https://patents.google.com/patent/US6821761B2) and human cathepsin S (CTSS; NCBI reference sequence: NP_004070.3) share 34% sequence identity.
