# Supplementary material for: Deletion of fatty acid amide hydrolase reduces lyso-sulfatide levels but exacerbates metachromatic leukodystrophy in mice
Source: J Biol Chem. 2021 Aug 8;297(3):101064. doi: 10.1016/j.jbc.2021.101064 (PMC8435702; doi:10.1016/j.jbc.2021.101064)
Supplement: Supplemental Table S1 [file mmc2.docx]

**Supporting Table 1**

| *enzyme*  *(species)* | *abbre-viation* | *EC number* | *cDNA source* | *vector (resistance)* |
| --- | --- | --- | --- | --- |
| dipeptidase I  (Homo sapiens) | DPEP1 | 3.4.13.19 | Invitrogen  clone ID 3846046 (IRAT) | pCMV-Sport6  (Amp) |
| aminoacylase  (Homo sapiens) | ACY1 | 3.5.1.14 | ImaGenes  IRAU p969D1218D  (IMAGE ID 2821665) | pOTB7  (Cam) |
| carboxypeptidase A  (Homo sapiens) | CPVL | 3.4.17.1 | ImaGenes  IRAT p970H0417D  (IMAGE ID 3888647) | pCMV-Sport6  (Amp) |
| carboxypeptidase C  (Homo sapiens) | SCPEP1 | 3.4.16.5 | ImaGenes  IRAT p970H09105D  (IMAGE ID 6137668) | pCMV-Sport6  (Amp) |
| cathepsin B  (Homo sapiens) | CTSB | 3.4.22.1 | ImaGenes  IRAT p970H04100D  (IMAGE ID 30334082) | pBluescriptR  (Amp) |
| cathepsin H  (Homo sapiens) | CTSH | 3.4.22.16 | ImaGenes  IRAU p969H0310D  (IMAGE ID 3349910) | pOTB7  (Cam) |
| cathepsin S  (Homo sapiens) | CTSS | 3.4.22.27 | ImaGenes  IRAU p969B0821D  (IMAGE ID 3610589) | pOTB7  (Cam) |
| cathepsin Z  (Homo sapiens) | CTSZ | 3.4.18.1 | ImaGenes  IRAU p969C02103D  (IMAGE ID 5018854) | pOTB7  (Cam) |
| acid ceramidase  (Homo sapiens) | ASAH1 | 3.5.1.23 | Invitrogen  clone ID 3923451 (IRAT) | pCMV-Sport6  (Amp) |
| neutral ceramidase  (Homo sapiens) | ASAH2 | --- | ImaGenes  IRCM p5012H084D  (IMAGE ID 40018929) | pCR-BluntII-TOPO  (Kan) |
| alkaline ceramidase-1  (Homo sapiens) | ACER1 | --- | ImaGenes  IRAT p970E02129D  (IMAGE ID 8327590) | pCR4-TOPO  (Amp) |
| alkaline ceramidase-2^(1)^  (Homo sapiens) | ACER2 | --- | ImaGenes  IRAT p970C07100D  (IMAGE ID 30528463) | pBluescriptR  (Amp) |
| alkaline ceramidase-3^(2)^  (Homo sapiens) | ACER3 | --- | ImaGenes  IRAT p970D0895  (IMAGE ID 3867010) | pCMV-Sport6  (Amp) |
| N-acylethanolamine-hydrolyzing acid amidase  (Mus musculus) | NAAA | 3.5.1.60 | Invitrogen  clone ID 3586255  (IRAV) | pCMV-Sport6  (Amp) |
| fatty acid amide hydrolase  (Homo sapiens) | FAAH | 3.5.1.99 | ImaGenes  IRAT p970F05127D  (IMAGE ID 8327409) | pCR4-TOPO  (Amp) |

^(1)^ cDNA was not full length ^(2)^ cDNA was not ACER3
