# Supplementary material for: Deletion of fatty acid amide hydrolase reduces lyso-sulfatide levels but exacerbates metachromatic leukodystrophy in mice
Source: J Biol Chem. 2021 Aug 8;297(3):101064. doi: 10.1016/j.jbc.2021.101064 (PMC8435702; doi:10.1016/j.jbc.2021.101064)
Supplement: Supplemental Table S2 [file mmc3.docx]

**Supporting Table 2**

Properties of established lysosomal proteins (according to Ref 17)

| **lysosomal protein** | **amidase activity^(1)^** | **endo-peptidase^(1)^** | **high expression in monocyte/ macrophage lineage^(2)^** | **substrate^(1)^** |
| --- | --- | --- | --- | --- |
| ABCA2 | no |  |  |  |
| ABCB9/TAP-like transporter | no |  |  |  |
| ATPase, V-H+ ~13 subunits | no |  |  |  |
| CD68 | no |  |  |  |
| CLC-7 | no |  |  |  |
| CLN3 protein | no |  |  |  |
| Cystinosin | no |  |  |  |
| LALP70 | no |  |  |  |
| LAPTM4 | no |  |  |  |
| LIMP-1/CD63/LAMP-3 | no |  |  |  |
| LIMP-2/LGP85 | no |  |  |  |
| Lipopolysaccharide-induced TNF factor LITAF | no |  |  |  |
| LYAAT-1 | no |  |  |  |
| Lysosome-associated membrane protein 1 (LAMP1) | no |  |  |  |
| Lysosome-associated membrane protein 2 (LAMP2) | no |  |  |  |
| Major facilitator superfamily domain containing 8 | no |  |  |  |
| Mucolipin | no |  |  |  |
| Nicastrin | no |  |  |  |
| NPC1 protein | no |  |  |  |
| Solute carrier family 17 | no |  |  |  |
| 1-O-acylceramide synthase | no |  |  |  |
| Acid ceramidase (ASAH1) | yes | no | yes | ceramide |
| Acid lipase | no |  |  |  |
| Alpha-galactosidase A | no |  |  |  |
| Alpha-L-iduronidase | no |  |  |  |
| Alpha-N-acetylgalactosaminidase | no |  |  |  |
| Alpha-N-acetylglucosaminidase | no |  |  |  |
| Arylsulfatase A | no |  |  |  |
| Arylsulfatase B | no |  |  |  |
| Beta-galactosidase | no |  |  |  |
| Beta-glucuronidase | no |  |  |  |
| Beta-hexosaminidase alpha chain | no |  |  |  |
| Beta-hexosaminidase beta chain | no |  |  |  |
| Beta-mannosidase | no |  |  |  |
| Carboxypeptidase, vitellogenic-like (CPVL) | yes | no | yes | (di)peptides |
| Cathepsin B (CTSB) | yes | no | yes | peptides |
| Cathepsin D (CTSD) | yes | yes | yes | peptides |
| Cathepsin F (CTSF) | yes | yes | no | peptides |
| Cathepsin H (CTSH) | yes | no | yes | peptides |
| Cathepsin K (CTSK) | yes | yes | no | peptides |
| Cathepsin L (CTSL) | yes | yes | yes | peptides |
| Cathepsin O (CTSO) | yes | ? | nd | (poorly characterized) |
| Cathepsin S (CTSS) | yes | yes | yes | peptides |
| Cathepsin Z (CTSZ) | yes | no | yes | peptides |
| CLN5 protein | no |  |  |  |
| Deoxyribonuclease II | no |  |  |  |
| Dipeptidyl-peptidase I | yes | yes | yes | peptides |
| Galactocerebrosidase | no |  |  |  |
| Gamma-glutamyl hydrolase | yes | no | no | gamma-glutamates |
| Glycosylasparaginase | no |  |  |  |
| GM2 activator | no |  |  |  |
| Hyaluronidase | no |  |  |  |
| Iduronate 2-sulfatase | no |  |  |  |
| IFNγ inducible protein 30 | no |  |  |  |
| Legumain | yes | yes | yes | peptides |
| Lysosomal alpha-glucosidase | no |  |  |  |
| Lysosomal alpha-mannosidase | no |  |  |  |
| Lys. protect. prot. /Cathepsin A (SCPEP1)^(3)^ | yes | no | yes | peptides |
| Myeloperoxidase | no |  |  |  |
| N-acetylgalactosamine-6-sulfatase | no |  |  |  |
| N-acetylglucosamine-6-sulfatase | no |  |  |  |
| NPC2 protein | no |  |  |  |
| N-sulphoglucosamine sulphohydrolase/heparan N-sulfatase | no |  |  |  |
| Palmitoyl-protein thioesterase 1 | no |  |  |  |
| Palmitoyl-protein thioesterase 2 | no |  |  |  |
| Saposin | no |  |  |  |
| Sialic acid 9-O-acetylesterase | no |  |  |  |
| Sialidase 1 | no |  |  |  |
| Sialidase 4 | no |  |  |  |
| Sphingomyelin phosphodiesterase | no |  |  |  |
| Tartrate-resistant acid phosphatase | no |  |  |  |
| Tissue alpha-L-fucosidase | no |  |  |  |
| Tripeptidyl-peptidase I | yes | yes | yes | peptides |
| Lysosomal acid phosphatase | no |  |  |  |
| Glucocerebrosidase | no |  |  |  |

^(1)^according to https://www.brenda-enzymes.org/

^(2)^according to http://genevisible.com

^(3)^recommended name is Carboxypeptidase C
