# Supplementary material for: Deletion of fatty acid amide hydrolase reduces lyso-sulfatide levels but exacerbates metachromatic leukodystrophy in mice
Source: J Biol Chem. 2021 Aug 8;297(3):101064. doi: 10.1016/j.jbc.2021.101064 (PMC8435702; doi:10.1016/j.jbc.2021.101064)
Supplement: Supplemental Table S3 [file mmc4.docx]

**Supporting Table 3**

Properties of potentially novel soluble lysosomal proteins (according to Ref 17)

| **lysosomal protein** | **amidase activity^(1)^** | **endo-peptidase^(1)^** | **high expression in monocyte/ macrophage lineage^(2)^** | **substrate^(1)^** |
| --- | --- | --- | --- | --- |
| Acyloxyacyl hydrolase | no |  |  |  |
| Arylsulfatase G | no |  |  |  |
| Arylsulfatase K | no |  |  |  |
| N-acylsphingosine amidohydrolase 2 (ASAH2) | yes | no | no | ceramide |
| N-acylethanolamine-hydrolyzing acid amidase (NAAA) | yes | no | yes | acylethanolamines |
| Biotinidase precursor | no |  |  |  |
| Cat eye syndrome critical region 1 | no |  |  |  |
| Clusterin (apoJ) | no |  |  |  |
| Cellular repressor of E1A-stimulated genes | no |  |  |  |
| Deoxyribonuclease 1 | no |  |  |  |
| Mammalian ependymin related protein 1 | no |  |  |  |
| Epididymis-specific alpha-d-mannosidase | no |  |  |  |
| ER aminopeptidase 1 (ERAP1) | yes | no | no | peptides |
| Hypothetical protein FLJ22662 | ? |  |  |  |
| Plasma alpha-L-2-fucosidase | no |  |  |  |
| Interleukin-4-induced gene 1 | ? |  |  |  |
| Mannose-6-phosphate protein P76 | ? |  |  |  |
| Phospholipase D3 | no |  |  |  |
| Procollagen-lysine 1,2-oxoglutarate 5-dioxygenase 1 | no |  |  |  |
| Protein O-fucosyltransferase 1 | no |  |  |  |
| Protein O-fucosyltransferase 2 | no |  |  |  |
| Prostaglandin-H2 D-isomerase | no |  |  |  |
| Pancreatic ribonuclease | no |  |  |  |
| Ribonuclease 6 | no |  |  |  |
| Ribonuclease T2 | no |  |  |  |
| Serine carboxypeptidase 1 (SCPEP1)^(3)^ | yes | no | yes | peptides |
| Neuroserpin | no |  |  |  |
| Acid sphingomyelinase-like phosphodiesterase 3A | no |  |  |  |
| Microsomal stress 70 protein ATPase | no |  |  |  |
| Sulfatase modifying factor 2 | no |  |  |  |

^(1)^according to https://www.brenda-enzymes.org/

^(2)^according to http://genevisible.com

^(3)^identical to lysosomal protective protein/cathepsin A (carboxypeptidase C) from list of established lysosomal enzymes (Supporting table 2)
