# Supplementary material for: Deletion of fatty acid amide hydrolase reduces lyso-sulfatide levels but exacerbates metachromatic leukodystrophy in mice
Source: J Biol Chem. 2021 Aug 8;297(3):101064. doi: 10.1016/j.jbc.2021.101064 (PMC8435702; doi:10.1016/j.jbc.2021.101064)
Supplement: Supplemental Table S4 [file mmc5.docx]

**Supporting Table 4**

List of selected lysosomal enzymes with possibly matching endo-N-deacylase activity

| **enzyme** |
| --- |
| Acid ceramidase (ASAHI)^(1)^ |
| Carboxypeptidase, vitellogenic-like (CPVL)^(1)^ |
| Cathepsin B (CTSB)^(1)^ |
| Cathepsin H (CTSH)^(1)^  Cathepsin S (CTSS)^(2)^ |
| Cathepsin Z (CTSZ)^(1)^ |
| Lysosomal protective protein/Cathepsin A (carboxypeptidase C) (SCPEP1)^(1)^ |
| N-acylethanolamine-hydrolyzing acid amidase (NAAA)^(1)^ |

^(1)^amidase activity, no endopeptidase activity, high expression in monocyte/macrophage lineage

^(2)^sequence similarities with prokaryotic SCDase (see Supporting Fig. 1B)
